# Inference of the drivers of collective movement in two cell types: *Dictyostelium* and melanoma

**DOI:** 10.1098/rsif.2016.0695

**Published:** 2016-10

**Authors:** Elaine A. Ferguson, Jason Matthiopoulos, Robert H. Insall, Dirk Husmeier

**Affiliations:** 1 Institute of Biodiversity, Animal Health and Comparative Medicine, College of Medical, Veterinary and Life Sciences, University of Glasgow, Glasgow, UK; 2 School of Mathematics and Statistics, College of Science and Engineering, University of Glasgow, Glasgow, UK; 3 Cancer Research UK Beatson Institute, Glasgow, UK

**Keywords:** advection–diffusion–reaction, self-generated gradients, collective cell movement, model selection, bootstrapping, widely applicable information criterion

## Abstract

Collective cell movement is a key component of many important biological processes, including wound healing, the immune response and the spread of cancers. To understand and influence these movements, we need to be able to identify and quantify the contribution of their different underlying mechanisms. Here, we define a set of six candidate models—formulated as advection–diffusion–reaction partial differential equations—that incorporate a range of cell movement drivers. We fitted these models to movement assay data from two different cell types: *Dictyostelium discoideum* and human melanoma. Model comparison using widely applicable information criterion suggested that movement in both of our study systems was driven primarily by a self-generated gradient in the concentration of a depletable chemical in the cells' environment. For melanoma, there was also evidence that overcrowding influenced movement. These applications of model inference to determine the most likely drivers of cell movement indicate that such statistical techniques have potential to support targeted experimental work in increasing our understanding of collective cell movement in a range of systems.

## 1. Introduction

Collective movements are important in many cell systems, affecting processes of considerable medical interest, including wound healing, the immune response and the spread of cancers. Cell movements can have random (diffusive) and directional components. Chemotaxis, the movement of cells up or down spatial gradients in the concentrations of chemicals (chemoattractants or chemorepellants), is the process underlying many of the directional cell movements that we observe [1]. The chemical gradients to which cells respond can result from chemicals diffusing from a local source, which is typically formed by either the cells themselves or nearby cells of a different type releasing chemicals into the environment. An example of local source gradient generation is the suggested mechanism by which macrophages promote metastasis of breast tumours; the tumour cells release an attractant for macrophages, which chemotax towards the tumour and release an attractant for the tumour cells, encouraging their migration away from the primary tumour [2]. Chemical gradients may also result from local sinks, which are typically caused by cells depleting a chemical from their environment. Recent studies have suggested that cell movements caused by chemotactic gradients that cells self-generate by depletion may be common to a wide range of cell types [3–7]. Cell movements resulting from diffusion and chemotaxis may additionally be influenced by density-dependent effects. If cells are in a tightly packed environment, then they may restrict each other's abilities to move in response to stimuli. The process of contact inhibition of locomotion (CIL), which occurs in many cell types and forces cells to change direction when they contact one another [8], also has a more pronounced effect at high density.

Identification of the drivers of movement in a particular cell system is a crucial step in understanding how we might influence that system through new medical interventions, such as the use of chemical-releasing implants to disrupt chemotactic gradients responsible for cancer cell migration [9,10]. However, without any prior knowledge, identifying movement drivers experimentally can be a long process. Mathematical models offer a potential solution. By fitting sets of candidate cell movement models to data from cell systems, and then carrying out model comparison to identify the best model, we can get an indication of what mechanisms are most likely to be driving movement in those systems. This information could then be used to guide experimental work, to confirm the existence of these mechanisms.

Since the development of the Keller–Segel model to describe the aggregation of *Dictyostelium discoideum* cells in 1970 [11], a large body of work has emerged on the modelling of cell movement mechanisms using partial differential equations (PDEs); see Hillen & Painter [12] for a guide to these models. However, we are unaware of any attempts to formally fit these models to cell movement data and infer movement drivers through model comparison. A possible reason for this is computation. The PDEs involved are of the advection–diffusion–reaction type, describing spatio-temporal changes in the distribution of cells as a result of random cell movements (diffusion), directional movements through chemotaxis (advection) and changes in the numbers of cells through cell division and death (reaction). PDEs with the level of complexity and flexibility required to simulate realistic cell movements typically have to be solved and optimized numerically, which incurs high computational costs. Numerical solution of the models also introduces error, and when advection is strong relative to diffusion, this error can manifest as oscillations in the modelled cell density. When severe, these instabilities can cause the model solver to fail, halting parameter optimization prematurely [13]. Inference is further complicated by the presence of local likelihood optima that can trap optimization algorithms, and a lack of data on variables such as chemical concentrations in space and time.

In this study, we describe six candidate models for cell movement that incorporate various biological hypotheses, including chemotaxis up self-generated gradients, repulsive and attractive interactions between the cells, and interference effects due to cell crowding. We then develop a methodology for fitting these models to data that attempts to overcome the associated challenges outlined above. This methodology is tested on data from movement assays for cells of two different types: *Dictyostelium discoideum* and human melanoma. *Dictyostelium* is an amoeba that is frequently used as a model organism for eukaryotic cell movement [14] and is known to chemotax in order to find bacteria when feeding and to aggregate when starved [15]. Melanoma is a cancer that is made particularly aggressive by the rapidity with which it spreads, with the risk of metastasis increasing sharply with the thickness of the tumour [16,17]. Given that metastasis is the primary cause of human cancer deaths [18], understanding why these cells move is important. Recent work has suggested that migration of melanoma cells away from the primary tumour is driven by the tumour becoming large enough to create a local gradient in the chemoattractant lysophosphatidic acid (LPA) through depletion [3]. Here, we attempt to draw conclusions about the drivers of movement in these cell types, under the conditions of the particular movement assays studied, by applying our model fitting methodology to data from these assays and carrying out model comparison. Note that the major driver of movement in the two datasets, a self-generated gradient in attractant, has already been determined experimentally [3,7], so that the ability to identify this key mechanism provides a useful test for our inference scheme. Self-generated gradients are important in driving movement in a range of systems [3–7], and the development of model selection methods that can detect this driver is, therefore, particularly desirable. Other processes that could be playing a more minor role in producing the movement patterns observed in our data, such as overcrowding or chemical interactions between the cells, have been less exhaustively tested for, and so we also test for these within our set of candidate models.

## 2. Data

Data on the collective movement of *Dictyostelium* cells during an under-agarose assay [19] were collected by Tweedy *et al.* [7]. The agarose under which the cells moved contained folate, a chemoattractant that the cells can deplete from their environment, at an initially homogeneous concentration of 10 µM. Under these conditions, *Dictyostelium* cells create a gradient in folate through depletion, and then collectively move up this gradient [7].

A similar dataset on the collective movement of melanoma cells was collected by Muinonen-Martin *et al*. [3]. Here, the migration of the cells was observed between two wells connected by a bridge in a direct visualization chamber [20] that was homogeneously filled with 10% FBS (fetal bovine serum). It was previously determined experimentally that collective movement in this case is primarily driven by a self-generated gradient in LPA, a component of FBS that can be depleted by the melanoma cells [3].

**Figure 1.**
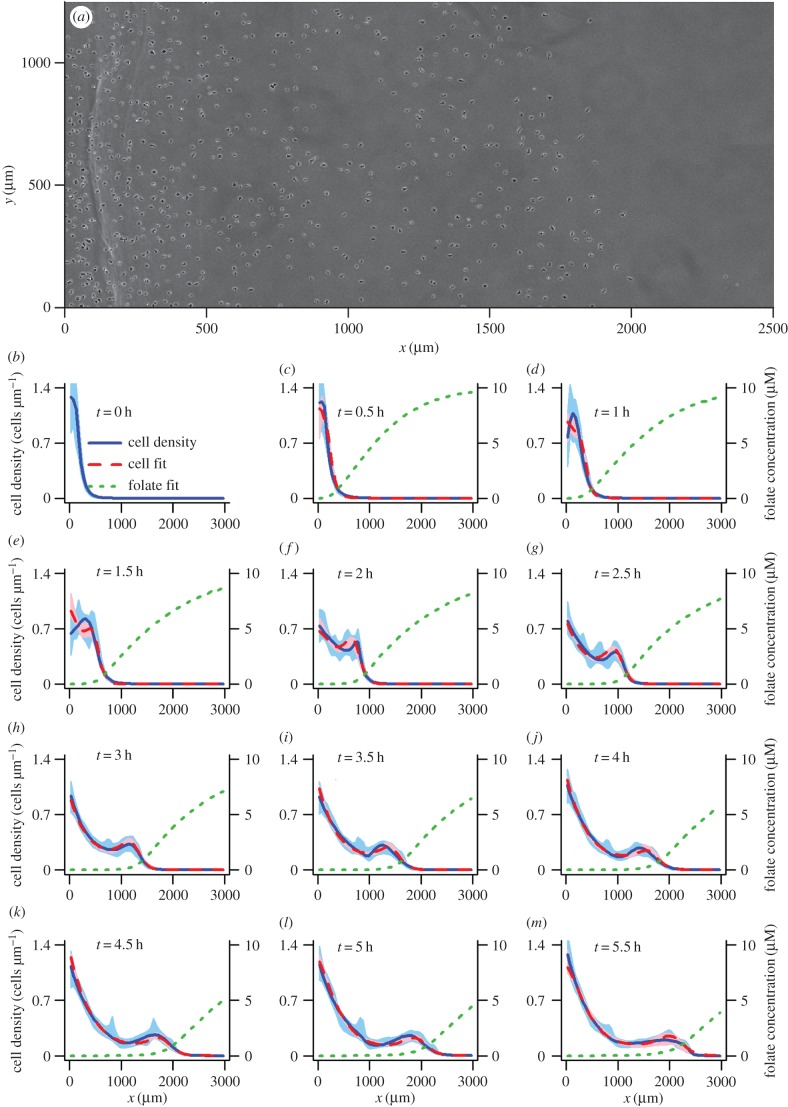
(*a*) Image taken 4 h into the *Dictyostelium discoideum* cell movement assay (see (*j*) for corresponding cell density estimate). (*b–m*) Cell distributions obtained every half hour using logspline density estimation [21–23] in the *x*-dimension are shown by blue lines, with 95 percentile intervals obtained using 10 000 bootstrap samples of the data indicated by blue shaded areas. Cell distributions produced by the best model (the receptor saturation model, table 1) for this dataset, using the optimized parameters from the bootstrap optimization that gave the highest value of the maximum weighted log-likelihood (equation (4.4)), are shown by dashed red lines. The corresponding folate distributions predicted by this model are indicated by green dotted lines. Pink shaded areas show the 95 percentile interval for the modelled cell density, based on 200 samples from the pseudo-posterior.

**Figure 2.**
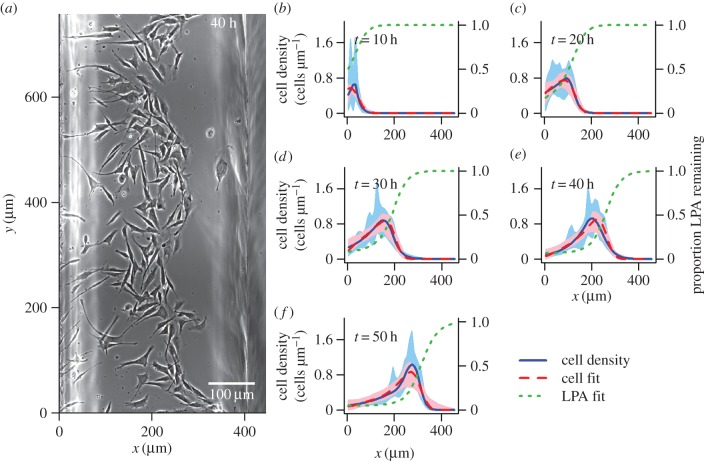
(*a*) Image taken 40 h into the melanoma cell movement assay (see (*e*) for corresponding cell density estimate). (*b–f*) Cell distributions obtained every half hour using logspline density estimation [21–23] in the *x* dimension are shown by blue lines, with 95 percentile intervals obtained using 10 000 bootstrap samples of the data indicated by blue shaded areas. Cell distributions produced by the best model (the overcrowding model, table 1) for this dataset, using the optimized parameters from the bootstrap optimization that gave the highest value of the maximum weighted log-likelihood (equation (4.4)), are shown by dashed red lines. The corresponding LPA distributions predicted by this model are indicated by green dotted lines. Pink shaded areas show the 95 percentile interval for the modelled cell density, based on 200 samples from the pseudo-posterior.

*Dictyostelium* cells move more rapidly than melanoma cells, so the *Dictyostelium* dataset covers both a larger spatial distance (approx. 2500 µm compared to approx. 400 µm), and a shorter time frame (5.5 h compared to 50 h) than the melanoma dataset. We extracted the cell coordinates manually from microscopy images at half-hour time intervals for *Dictyostelium* and 10 h intervals for melanoma (see the electronic supplementary material, supplement A for an investigation of the error involved in the extraction process). The cells were initialized in a linear group along the *y*-axis in both assays. Therefore, we were primarily interested in movement in the perpendicular direction, along the *x*-axis, and reduced the data to one spatial dimension (*x*) for our analyses. One-dimensional logspline density estimates [21–23] were used to visualize the spread of the cells up the spatial axis for both *Dictyostelium* (figure 1) and melanoma (figure 2).

**Table 1.** WAIC (equation (5.1)) comparison of the six candidate models for both datasets. Standard errors (in brackets) were calculated as described in the electronic supplementary material, Supplement F.

model	WAIC	
	*Dictyostelium*	melanoma
diffusion	88 367.1 (0.10)	5985.5 (0.03)
basic	87 970.7 (0.77)	5736.2 (7.70)
receptor saturation	87 631.2 (0.39)^a^	5719.9 (3.10)
interaction	87 636.8 (0.44)	5743.1 (2.08)
overcrowding	87 648.0 (0.44)	5712.2 (1.85)^a^
full	87 646.3 (0.47)	5739.6 (2.25)

^a^The best model for each dataset (i.e. the model with the lowest WAIC value).

Spatio-temporal variation in the concentration of the chemoattractants, folate and LPA, was unmeasurable during the assays. Therefore, we treated these concentrations as latent variables during model fitting.

## 3. Models

All of the cell movement models considered in this study involve one-dimensional advection–diffusion–reaction PDEs of the form
3.1
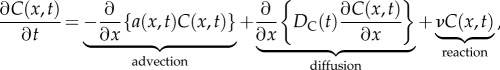
where *t* is time, *x* is space and *C*(*x*, *t*) is cell density. A positive or negative value of the advection coefficient *a*(*x*, *t*) leads to directional movement towards higher or lower *x*, respectively. The diffusion coefficient 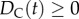 describes the rate at which cells spread out from high- to low-density areas via randomly directed movements, and the reaction term describes exponential growth of the cell population through cell division at rate 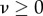.

We investigated six different advection coefficients, each representing a hypothesis for the drivers of cell movement. Our *diffusion model* assumes that cell movement is simply random, with no directional movement component, i.e.
3.2
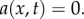


Directional movement up a spatial gradient in the concentration of an attractant *A*(*x*, *t*) is described in the *basic model*:
3.3
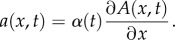
Here, the rate of advective cell movement depends on both the strength of the gradient in *A*(*x*, *t*) and the magnitude of the parameter 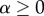. The attractant concentration is modelled through a second PDE:
3.4
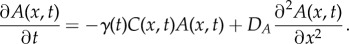


This function allows the cells to create self-generated gradients in *A*(*x*, *t*) through local depletion in proportion with their density and the remaining level of attractant, at a rate determined by 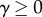. The parameter *D_A_* describes the constant rate at which attractant diffuses in the medium.

While our basic model (equation (3.3)) assumes that the ability of the cells to chemotax up a gradient in attractant is influenced only by the steepness of the gradient, it has been shown that chemotaxis also depends on the concentration of chemoattractant in a cell's local environment [24]. This dependency is a result of receptor saturation. Cells detect spatial gradients in chemicals through the resulting gradients in the occupancy of their surface receptors for those chemicals. When the background chemoattractant concentration is high, a cell's receptors can become saturated, so that an underlying chemotactic gradient fails to produce a detectable gradient in receptor occupancy, preventing accurate chemotaxis. In our *receptor saturation model*, we replace the chemoattractant gradient of the basic model (equation (3.3)) with a gradient in receptor occupancy, calculated according to the single-site equilibrium dissociation equation, where *K*_d_ is the dissociation constant, as follows:
3.5
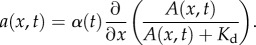
Cell movement may be influenced by attractive or repulsive chemical interactions between the cells. In the *interaction model*, we incorporate these behaviours by allowing the cells to move directionally in response to gradients in their own density, in addition to the gradient in receptor occupancy for *A*(*x*, *t*):
3.6
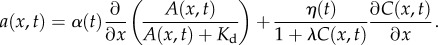


Here, a negative *η* indicates repulsion between the cells and a positive *η* indicates attraction. The strength of the interaction is reduced at high cell densities through the parameter 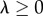. This feature is intended to mimic the effect of saturation of the cell receptors for the chemical involved in the interaction; at high cell density, we would expect higher concentrations of the chemical released by the cells, leading to saturation effects that reduce the ability of the cells to detect and migrate in response to the conspecific density gradient. Keller & Segel [11] previously proposed a method for modelling cell interactions, in which the cells respond directly to the interaction chemical, the production and decay of which is modelled through an additional PDE. Our more indirect approach, where the cells instead respond to their own density gradient, has the advantages that it requires fewer new parameters, which simplifies model fitting, and it avoids the need to make an assumption about the unknown initial distribution of the interaction chemical.

It is expected that the ability of cells to move freely will be reduced at high density, both because tight packing of cells means that there is physically less space for them to move into, and because more contact between cells occurs at high density, meaning that the effects of CIL [8] will be more evident. We incorporate these effects into the receptor saturation model (equation (3.5)) to produce the *overcrowding model*:
3.7
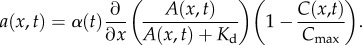
The new term in the advection coefficient, which is derived by Hillen & Painter [12], causes advection up the gradient in receptor saturation to slow as cell density approaches its maximum value *C*_max_.

Finally, our *full model* combines the effects of receptor saturation, cell interactions and overcrowding effects, with the advection coefficient:
3.8
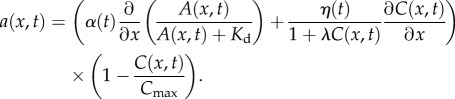


**Figure 3.**
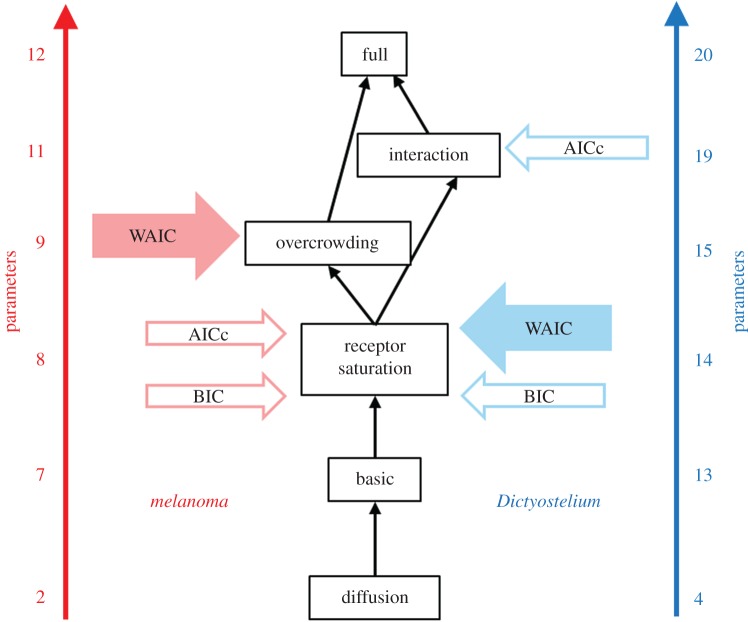
Graph illustrating the relationships between our candidate models (described in §3). Wherever two models occupy adjacent nodes, it is possible for the more complex model (with the greater number of parameters) to be reduced to the less complex one by constraining parameters. The number of parameters given for each model is based on a degree of one for the polynomials describing the time-varying parameters for melanoma, and a degree of three for *Dictyostelium* (see the electronic supplementary material, tables S1 and S2). For each dataset, the models preferred by WAIC, AICc and BIC (see table 1; and electronic supplementary material, tables S3 and S4) are indicated with arrows.

Note that all of our models are nested within the full model as illustrated in the model relational graph of figure 3.

Four of our model parameters *α*, *D*_C_, *γ* and *η*, which relate to cell advection and diffusion rates, and the rate of depletion of chemoattractant, are permitted to vary in time to allow for changes in cell behaviour over the course of the assays. These temporal dependencies were introduced by modelling the parameters as polynomial functions of time, which were exponentiated for those parameters that were restricted to positive values (*α*, *D*_C_ and *γ*). The degrees of the polynomial functions were selected as described in §5.

## 4. Likelihood calculation

For a given dataset, model and set of parameters ***θ***, we obtained spatio-temporally varying functions describing cell density *C*(*x*, *t*) and attractant concentration *A*(*x*, *t*) by solving the PDEs numerically using the method of lines [25,26] (electronic supplementary material, Supplement B.1). For melanoma, there were no cells in the observation region at *t* = 0, so we used initial conditions of *C*(*x*, 0) = 0 and *A*(*x*, 0) = 1 (100% of the initial concentration of the attractant (LPA) remaining in the serum). For *Dictyostelium*, where some cells had already moved into the observation area at *t* = 0, the initial distribution of cells was obtained by applying logspline density estimation [21–23] to the cell location data. We assumed a sigmoidal function for the unobserved initial distribution of the attractant for *Dictyostelium* (folate), the parameters of which were estimated along with the model parameters. Increases in the total number of cells due to cell division were relatively minor over the time period of interest for *Dictyostelium*, so we set *ν* (see equation (3.1)) to zero. For melanoma, the value of *ν* was estimated from the data as described in the electronic supplementary material, Supplement B.3. In both datasets, large numbers of cells moved into the observation region via the left boundary, and we captured these movements by introducing a cell flux across this boundary, which was equal to the rate of change in the number of cells observed in the region minus the rate of change in cell numbers due to cell division. Full details on our choices of boundary and initial conditions can be found in the electronic supplementary material, Supplement B.

The models were fitted to the cell locations at the *T* time points for each dataset. The raw observations 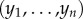 were, thus, each referenced by both a spatial location and time, i.e. 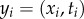. The total number of cells observed over the *T* time points was given by
4.1
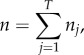
where *n_j_* was the number of cells observed at time point 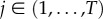
*.*

Following numerical integration of the model, the likelihood of ***θ*** can be calculated for each *y_i_* as
4.2
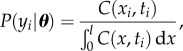
where *l* is the length of the modelled region. By summing over the *y_i_*, we could then obtain the total log-likelihood as
4.3
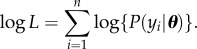
However, because the number of cells observed increases over time for both datasets, this standard log-likelihood will be biased towards producing a good fit at the end of the time period considered, potentially leading to a poorer match between model and data at the beginning of the time period. An alternative method that corrects for this bias is to weight each 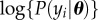 according to the total number of cells observed at the corresponding time point as follows:
4.4
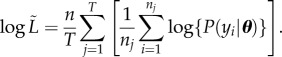


In this weighted log-likelihood calculation, the multiplication by *n*/*T* returns the value to the scale of the standard log-likelihood.

## 5. Model inference and comparison

For all models considered, it was necessary to infer both the model parameters and, for *Dictyostelium*, also the parameters of the sigmoidal distribution describing the unknown initial distribution of folate (see the electronic supplementary material, Supplement B.2). During inference, we used a lower bound of zero for the diffusion coefficient *D_A_* of LPA in the melanoma assay, while for *Dictyostelium*, we used the literature values for the diffusion coefficient of folate [27,28] to introduce more restrictive upper and lower bounds of 200 µm^2^ s^−1^ and 150 µm^2^ s^−1^, respectively, for *D_A_*. For both datasets, we set a lower bound for *C*_max_ that was equal to the maximum cell density value observed in the logspline density estimates obtained from the cell location data (blue lines in figures 1 and 2). We bounded the parameters *K*_d_ and *λ* below by zero, leaving them unbounded above. The parameters describing the initial folate distribution were given upper and lower bounds that prevented initial distributions known to be unrealistic (see the electronic supplementary material, Supplement B.2). The remaining parameters (*α*, *γ*, *η* and *D*_C_) were modelled as polynomial functions of time, which for *α*, *D*_C_ and *γ* were exponentiated to bound the functions below by zero. The coefficients of the polynomial functions were unbounded during model inference.

It was necessary to select the degrees of the polynomial functions used to describe our time-varying parameters. Ideally, we would do this by carrying out inference for each model on each dataset using a range of polynomial degrees for each of the parameters, and then applying model comparison to select the best combination of polynomial degrees for each model. However, inference for these models is computationally expensive, making such an exhaustive model comparison infeasible. We instead proceeded by fitting our most complex model (the full model, equation (3.8)) to each of our datasets by maximizing the weighted log-likelihood (equation (4.4); see the electronic supplementary material, Supplement C for details on the maximization procedure), and gradually increasing the degree of the polynomials, always keeping the degree the same for all time-varying parameters in the model. We stopped increasing the polynomial degree when we found no further improvement in the values of two model comparison statistics: AICc (the Akaike information criterion corrected for small sample sizes) [29,30] and BIC (Bayesian information criterion) [31]. Once we had used this maximum weighted log-likelihood approach to obtain the optimal polynomial degree for the time variance of the parameters for each dataset, we carried out inference for the full set of six candidate models, using the more computationally costly, but more reliable, pseudo-Bayesian approach described below, always using the previously selected polynomial degree.

The use of a Bayesian approach to obtain a posterior distribution of the parameters provides access to WAIC (widely applicable information criterion) [32]; a recently developed model comparison statistic that makes fewer assumptions than those commonly calculated from maximum-likelihood estimates (including AICc and BIC). The key improvement offered by WAIC is that it allows for the fact that some parameters might be poorly determined by the data (for details, see ch. 7 of Gelman *et al.* [33]). However, Markov chain Monte Carlo (MCMC) algorithms, the standard approach to obtaining a sample from the posterior, are intrinsically sequential, making them unable to exploit parallel computer clusters. This sequential nature of MCMC presents further problems for advection–diffusion models, as chains can break down or become trapped in regions of parameter space where unstable numerical solutions cause model solving algorithms to fail [13]. We avoided these issues by using the following method to obtain a pseudo-posterior for each of our models and datasets.

The cell location data were sampled with replacement for each time point involved in the fitting process to obtain many bootstrap datasets of the same size as the original ones. A maximization of the weighted log-likelihood (equation (4.4)) was then carried out for each model on each bootstrap dataset using an optimization algorithm (we found that the quasi-Newton BFGS algorithm performed well for the *Dictyostelium* data, while the Nelder–Mead algorithm was more effective at reaching high-likelihood parameter regions for the melanoma data). By optimizing on many re-samples of the data, we obtain many parameter sets that can be used as a proxy for a sample from the posterior distribution of the parameters, where there is an assumption of uniform prior distributions. The variance of this pseudo-posterior is driven by the uncertainty in the data, which is introduced through the bootstrapping procedure. Similar approaches to obtaining a pseudo-posterior have previously been applied by other authors; see, for example, Friedman *et al.* [34]. Note that this approach to inference is computationally costly, owing to the need to run many optimizations per model (we used 3000), but has advantages in being easily automated and parallelized. Additionally, any optimizations that fail due to numerical instability can simply be discarded and reinitialized.

As a result of the optimizer becoming trapped on local optima, we found that, for both datasets, the pseudo-posteriors obtained by this method tended to be multi-modal. We removed all but the highest likelihood peak in the pseudo-posteriors, as described in the electronic supplementary material, Supplement D, prior to using the pseudo-posteriors to calculate WAIC as
5.1
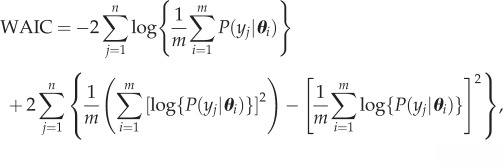
where *m* is the number of optimizations, 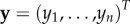 are the cell location data and ***θ****_i_* are the optimized parameter sets. To verify that WAIC approximated using a pseudo-posterior obtained by bootstrap sampling gives comparable results to the standard WAIC calculated by direct sampling from the true posterior, we carried out a test study that used both methods to select the order of a polynomial model fitted to independent benchmark data (electronic supplementary material, Supplement E). There was very close agreement between the WAIC values obtained using the two methods, suggesting that our pseudo-posterior is practically equivalent to the true posterior.

## 6. Results

Based on AICc and BIC, we selected a degree of three for the polynomial function describing the time variance of the parameters for *Dictyostelium*, and a degree of one for melanoma (electronic supplementary material, tables S1 and S2), suggesting that the *Dictyostelium* cells are changing their behaviour more rapidly than the melanoma cells.

For *Dictyostelium*, WAIC selects the receptor saturation model as the best model, while, for melanoma, the slightly more complex overcrowding model is preferred (table 1). While there are known issues with AICc and BIC—AICc can select overly complex models, whereas BIC typically selects overly simple models [35], and neither accounts for parameter uncertainty—that make them less reliable than WAIC, we also compared the models based on these simpler statistics to check for consistency (electronic supplementary material, tables S3 and S4). The difference between the model selected by WAIC and the models selected by AICc and BIC never exceeds a graph distance of one (figure 3).

For both datasets, the selected models produce very good visual agreement with the data (figures 1 and 2). These fits are a vast improvement over those produced by the simple diffusion model (electronic supplementary material, figures S1 and S2), and also provide a clear improvement over the basic model (electronic supplementary material, figures S3 and S4); the inclusion of the receptor saturation effect appears to allow the models to better replicate the peaked cell front, which the basic model tends to smooth over.

**Figure 4.**
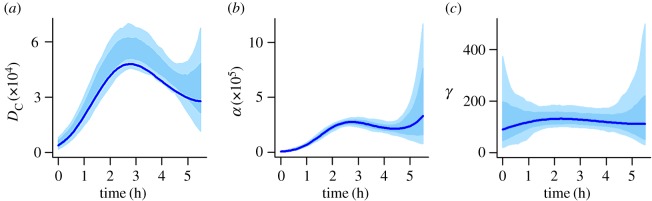
(*a*–*c*) Lines show the time-varying parameters for the best model (the receptor saturation model, table 1) for the *Dictyostelium* dataset, based on the mean of the pseudo-posterior obtained by many optimizations of the weighted log-likelihood (equation (4.4)) on bootstrap samples of the data. Shaded areas indicate 95 and 66 percentile intervals obtained from 200 samples from the pseudo-posterior. (Online version in colour.)

For *Dictyostelium*, the diffusion rate of the cells, *D*_C_, is estimated to first increase with time and then to decline again towards the end of the time period (figure 4*a*). The responsiveness of the *Dictyostelium* cells to the folate gradient, *α*, tends to increase over time (figure 4*b*), and the rate at which the cells deplete folate, *γ*, shows no clear trend (figure 4*c*). To investigate the importance of the time variance of each of these parameters in improving the fit of the selected model, we refitted the model multiple times by maximum weighted log-likelihood (see the electronic supplementary material, Supplement C), gradually replacing the time-varying parameters with constants and comparing these simplified models based on AICc and BIC (electronic supplementary material, table S5). We found that BIC selects only *α* and *D*_C_ to be time-varying parameters, suggesting that *γ* can be left time-invariant. The difference in AICc score between the model with all three time-varying parameters and the model with time-invariant *γ* is small. These findings are consistent with the trends in figure 4.

**Figure 5.**
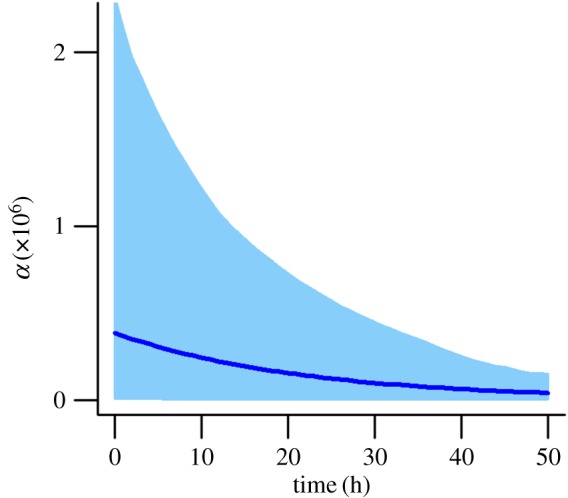
Time variance in *α* for the best model (the overcrowding model, table 1) for the melanoma dataset, based on the mean of the pseudo-posterior obtained by many optimizations of the weighted log-likelihood (equation (4.4)) on bootstrap samples of the data. The shaded area indicates the 66 percentile interval obtained from 200 samples from the pseudo-posterior. (Online version in colour.)

Carrying out a similar model selection for melanoma (electronic supplementary material, table S6), both AICc and BIC consistently suggest that the time dependence of *D*_C_ and *γ* can be removed, leaving *α* as the only time-varying parameter. A plot of the time dependence of *α* is given in figure 5, which shows a monotonically decreasing trend.

## 7. Discussion

Despite several decades of work developing mathematical models for collective cell movement, surprisingly little has been done to confront these models with data. Recent developments in both microscopy techniques and computer-intensive statistics are gradually removing the obstacles in this area. Here, we have begun exploring the technical challenges associated with carrying out statistical inference (comprising both parameter estimation and model selection) for PDE models using microscopy data on collective cell movement.

Our novel inference method, which involves running independent parameter optimizations on many bootstrap replicates of the data, was motivated by Friedman *et al.* [34], where it was referred to as a ‘poor man's’ approximation of the posterior distribution. In comparison with MCMC, our bootstrapping approach is easily automated and can be parallelized to spread the high computational cost over many processors. By generating a pseudo-posterior distribution, the bootstrapping approach also allows us to compute WAIC, which accounts for parameters that are poorly determined when penalizing model complexity, making it a more powerful and reliable model comparison statistic than AICc and BIC. This reduced penalty for poorly defined parameters may be why, in the melanoma case, WAIC selects a more complex model than AICc and BIC (figure 3). While we showed in a test study that obtaining WAIC from our pseudo-posterior gives good correspondence with the standard WAIC values calculated by sampling from the true posterior (electronic supplementary material, Supplement E), one issue that arose in our main study could potentially have led to a certain distortion in the approximations of the posterior distributions. This was that some optimizations failed due to instability in the numerical model solution at certain parameter combinations, which could mean that certain areas of parameter space are under-represented. These numerical instabilities are a known issue for advection–diffusion models that become evident when the Péclet number (the ratio of the advection coefficient to the diffusion coefficient, multiplied by the box length used when discretizing the PDE in space during numerical solution [36]) exceeds one. Our pseudo-posteriors, therefore, are limited to those regions where the numerical solutions of the models are relatively stable, and this may have led to them being different to the pseudo-posteriors that we would have obtained with accurate analytical solutions.

In addition to these limitations, statistical methods, on their own, are not able to identify a model with absolute certainty, as has been discussed, for example, in Burnham & Anderson [37]. This is a consequence of both sampling uncertainty, and the reliance of these methods on heuristic approximations (as discussed in the previous paragraph, or in Supplement C of the electronic supplementary material). However, statistical methods can identify those models that are most likely given current data, filtering out those that are unlikely to be correct and thus guiding future targeted experimental work to confirm the statistical findings. This makes model inference a useful tool, as narrowing down hypotheses using experiments alone is often made infeasible by the number and complexity of these hypotheses, and the cost of such experiments. We have critically assessed the reliability of the novel statistical procedures used here in three ways. First, we have tested them on two different model organisms: *Dictyostelium* and melanoma cells. Second, we compared our novel model selection scheme, based on WAIC, with two established asymptotic model selection criteria (AICc and BIC), and found that the models selected by these different statistics are never separated by a graph distance of more than one. This agreement between statistics is reassuring; we expect WAIC to provide a slight improvement on, but not a complete deviation from, the asymptotic results. Finally, while we lack complete *a priori* knowledge of the processes affecting cell movement in our datasets, we do have partial knowledge with which to validate the statistical results, as discussed in the next paragraph.

Through model inference and comparison, we have drawn a number of conclusions about the drivers of collective movement in assays for both *Dictyostelium* and melanoma cells. In both systems, the simple diffusion model is rejected as a description of the observed movement patterns in favour of our more complex models that incorporate directional movement in response to attractant gradients that are self-generated through depletion. This indication of the importance of the self-generated gradient mechanism shows agreement with experimental findings for melanoma [3], and experimental and simulation model results for *Dictyostelium* [7], that this mechanism is a key driver of the direction of chemotaxis in these systems. Confidence in the ability of our inference methods to identify the correct movement mechanisms is further increased by the fact that, for both cell types, we observe a substantial improvement of the receptor saturation model over the basic model (table 1). This agrees with the widely accepted concept that connection between extracellular signals and the intracellular mechanisms that drive cell migration occurs through cell-surface receptors. These receptors communicate to the inside of the cell by adopting two states, unoccupied and occupied; thus the only information seen by the motility machinery is the fractional occupancy of the receptors. At high receptor saturation, there can be very little difference in receptor occupancy between the front and rear of the cell. Incorporating receptor saturation led to our models being better able to replicate the form of the peak in cell density that marks the moving cell front. The receptor saturation effect causes this peak to become more defined, by causing the cells at the very front of the distribution, where attractant is most concentrated, to move more slowly than those directly behind, leading to a build-up of cells where the faster moving individuals meet the slower front-runners. Our fitting methods also allowed us to predict how the gradients in folate and LPA, on which we had no directly measured data, changed over the course of the assays. For *Dictyostelium*, the form of the predicted folate distribution gives a relatively close visual match to that measured experimentally by Tweedy *et al*. [7], using the same assay but with a higher initial folate concentration.

In addition to providing insights into the self-generated gradient mechanism, our model comparison study also suggests that an effect of cells blocking each other's movement when at high density (described in our overcrowding model) was evident in the melanoma data, but not in the *Dictyostelium* data. The primary reason for this difference may be that the cell densities in the *Dictyostelium* dataset never became high enough for overcrowding effects to exert an effect that our inference methods could detect; a visual comparison of images from the two datasets indicates that there are fewer direct contacts between the *Dictyostelium* cells (figure 1*a*) than between the melanoma cells (figure 2*a*). It is not completely clear how CIL would be expected to modify cells moving in a self-generated gradient, but this process is known to occur in neural crest cells [38]. As the melanocytes that mutate into melanoma cells develop from neural crest cells [39], it is likely that melanoma cells will also exhibit CIL, which may be a contributing factor to the selection of the overcrowding model for the melanoma dataset. Previous results simulated from an individual-based cell movement model suggested that CIL may also play a role in *Dictyostelium* movements in the system investigated here [7]. Our inability to detect this effect in *Dictyostelium* through a preference for the overcrowding model over the receptor saturation model may be a result of the loss of information incurred in moving from an individual-based modelling approach, where the movement path of each cell is known, to the population-based approach used in our study, where individual movement paths are not analysed.

Our model comparison finds no evidence for direct attractive or repulsive interactions between the cells for melanoma; a finding that is backed up by a lack of evidence for such conspecific interactions in the literature. For *Dictyostelium*, AICc suggests that such interactions may be important, but the other two comparison statistics (including the more reliable WAIC) place the interaction model second to the receptor saturation model (table 1; electronic supplementary material, table S3). Thus, while there may be some chemical communication between the *Dictyostelium* cells, its effect on the observed behaviour is not strong enough to be reliably detected. Vegetative *Dictyostelium* cells are known to secrete and respond to chemorepellents, but these appear to act over short time scales (minutes rather than hours) and ranges, so that repulsive interactions are not found to be important over the time frame and distances involved in our assay [40,41]. As *Dictyostelium* is well known for exhibiting aggregative interactions when exposed to prolonged starvation conditions [15], a shift in preference towards the interaction model may have been observed had we run the cell movement assay for a longer time period, or used *Dictyostelium* cells that were at a later stage in their development.

We found evidence in both of our datasets for changes in cell behaviour over time (figures 4 and 5; electronic supplementary material, tables S1 and S2). The diffusion coefficient for *Dictyostelium* is estimated to be low at the beginning of the assay (figure 4*a*), which may be a result of most of the cells still being in the process of transitioning under the gel at this stage. During this transition, the cells experience resistance from the gel [19], which will reduce the speed of diffusion. The diffusion rate increases once the cells have successfully moved under the gel, but then declines again towards the end of the time period, which may be a result of both starvation [42] and the cells changing their mode of motility from predominantly random movement towards chemotaxis, which is strong at the end of the time period (figure 4*b*). The chemotactic response of the *Dictyostelium* cells to the folate gradient increases over time. Slow initial chemotaxis may again be a result of the cells still adapting to move under the gel, while starvation may contribute to the subsequent increase in the efficiency of chemotaxis; starvation results in increasing polarity of the cells, leading to greater persistence in their direction of movement [43]. It is also possible that the decreased random movement and increase in chemotaxis are caused by repression of macropinocytosis, which is important for feeding but incompatible with chemotaxis [44]. The production of folate deaminase (the enzyme responsible for folate depletion) by *Dictyostelium* has previously been found to increase over time in response to folate exposure [45]. However, we find no evidence for this trend in the rate with which our cells deplete folate (figure 4*c*). It is possible that this increase in enzyme production had already occurred by the time the first image was obtained, over an hour after the cells were added to the system, and was, therefore, not detectable in the data. For melanoma, only the chemotactic responsiveness of the cells shows a temporal trend, declining over the course of the assay (figure 5). This decline could be caused by cells being imperfectly maintained during the longer assay conditions, or by endocytosis and degradation of the LPA receptor, which is a universal behaviour [4].

To conclude, we have developed an inference methodology that overcomes many of the computational difficulties associated with fitting a set of candidate PDE models for cell movement to data. We have applied these methods to data from two systems: one involving *Dictyostelium*, a well-studied model organism in this field, and the other involving human melanoma, a cancer made particularly aggressive by its rapid spread. Through model comparison, we have successfully drawn conclusions about the drivers of movement in these systems, many of which are in agreement with previous experimental and modelling work, and, thus, offer a validation of our inference methods. Our study systems here are relatively simple in comparison with the levels of complexity often observed *in vivo*, where multiple cell types may be interacting within a considerably more complex environment. However, they are nonetheless examples of real cell movement behaviour, one of which is of great medical relevance, in which we have been able to detect the presence of self-generated chemotactic gradients; a movement driver recently found to be important in many systems, including *in vivo* [3–7]. This success is an encouraging first step, indicating that model inference has the potential to support targeted experimental work in increasing our understanding of collective cell movement in a range of systems.

## Supplementary Material

Supplementary Information
