# Supplementary material for: Inference of the drivers of collective movement in two cell types: Dictyostelium and melanoma
Source: J R Soc Interface. 2016 Oct;13(123):20160695. doi: 10.1098/rsif.2016.0695 (PMC5095226; doi:10.1098/rsif.2016.0695)
Supplement: Supplementary Information [file rsif20160695supp1.pdf]

Supplementary Information for "Inference of the drivers of collective movement in two cell types: Dictyostelium and melanoma"

Elaine A. Ferguson[1*], Jason Matthiopoulos[1], Robert H. Insall[2] and Dirk Husmeier[3]

[1] Institute of Biodiversity, Animal Health and Comparative Medicine, College of Medical, Veterinary and Life Sciences, University of Glasgow; [2]Cancer Research UK Beatson Institute; [3]School of Mathematics and Statistics, College of Science and Engineering, University of Glasgow

*e.ferguson.2@research.gla.ac.uk

Supplement A: Observation noise

The models that we fitted to the cell location data did not incorporate the observation noise that results from imperfect extraction of the cell coordinates from the microscopy images. To investigate whether this was a reasonable assumption, we extracted these coordinates from the *Dictyostelium* dataset on two independent occasions, with an interval of a year between them. Pairing each cell location $\tilde{x}_i$ in one of these extractions with the nearest cell location $\tilde{\tilde{x}}_i$ from the other extraction showed that there is close agreement between the two (Fig. A1). We can assume that the observation noise follows a Gaussian distribution with a mean of zero and a variance that can be estimated from the paired data points as:

$$\sigma^2 = \frac{1}{NT}\sum_{i=1}^{N}\sum_{k=1}^{T}\left(\tilde{x}_i\left(t_k\right) - \tilde{\tilde{x}}_i\left(t_k\right)\right)^2 = 11.1\mu m^2 \quad \text{(A3)}$$

From this we estimate a standard deviation of $\sigma = 3.3\mu m$ for the observation noise. Numerical solution of the partial differential equations for *Dictyostelium* was carried out on a spatial grid with a resolution of $50\mu m$, an order of magnitude larger than σ. As a result, accounting for σ in our models is unnecessary; our assumption of no observation noise is reasonable.

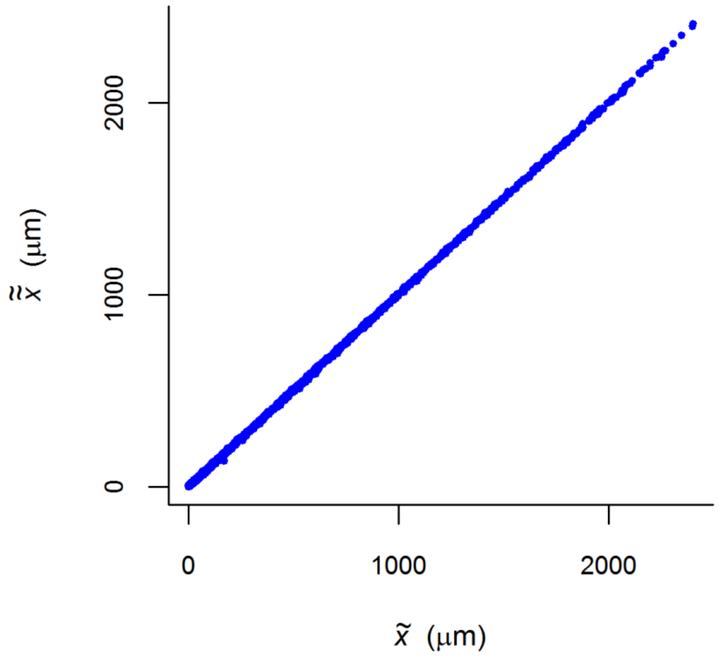

Figure A1. Scatterplot showing two independent extractions $\tilde{x}_i$ and $\tilde{\tilde{x}}_i$ of the spatial coordinate of each cell in the *Dictyostelium* dataset.

Supplement B: Numerical model solution

B.1. The method of lines

We numerically solved the partial differential equations on which our models were based using the method of lines [1,2]. This involved discretising our spatial region of interest of length l into equal-sized boxes, so that changes in cell density and attractant concentration in these boxes through time could be described as a system of ordinary differential equations (ODEs).

The basic form of our cell movement PDEs (equation (3.1)) can be rewritten as:

$$\begin{aligned}\frac{\partial C(x,t)}{\partial t} &= -\frac{\partial}{\partial x}\left\{a(x,t)C(x,t) - D_C(t)\frac{\partial C(x,t)}{\partial x}\right\} + \nu C(x,t) \\ &= -\frac{\partial Flux_C(x,t)}{\partial x} + \nu C(x,t)\end{aligned} \tag{B1}$$

where $Flux_C$ is the cell flux, which describes the net movement of cells up the spatial axis if positive and down the spatial axis if negative. Our one-dimensional spatial regions of interest were divided into boxes of length $\Delta x = 50\mu m$ for *Dictyostelium* and $\Delta x = 10\mu m$ for melanoma, allowing cell density changes in box $i \in (1,\ldots,B)$ to be described:

$$\frac{dC^i(t)}{dt} = -\frac{Flux_C^{i,i+1}(t) - Flux_C^{i-1,i}(t)}{\Delta x} + \nu C^i(t) \tag{B2}$$

where $Flux_C^{i-1,i}$ describes the cell flux between boxes $i-1$ and i. The cell fluxes across the boundaries of the modelled region were specified as described below in Supplement B.4. Fluxes over the region's internal box boundaries were obtained by approximating the spatial derivatives by finite differencing. For example, given equations (3.3, B1), $Flux_C$ for the basic model is:

$$Flux_C(x,t) = \alpha(t)\frac{\partial A(x,t)}{\partial x}C(x,t) - D_C(t)\frac{\partial C(x,t)}{\partial x} \tag{B3}$$

and the $Flux_C^{i-1,i}$ are estimated by:

$$Flux_C^{i-1,i}(t) = \alpha(t)\frac{A^i(t) - A^{i-1}(t)}{\Delta x}C^{i-1}(t) - D_C(t)\frac{C^i(t) - C^{i-1}(t)}{\Delta x} \tag{B4}$$

For those models incorporating the attractant $A(x,t)$, the additional attractant PDE (equation (3.4)) can, like the cell PDE (equation (B1)), be rewritten in terms of fluxes:

$$\begin{aligned}\frac{\partial A(x,t)}{\partial t} &= -\gamma(t)C(x,t)A(x,t) - \frac{\partial}{\partial x}\left\{-D_A\frac{\partial A(x,t)}{\partial x}\right\} \\ &= -\gamma(t)C(x,t)A(x,t) - \frac{\partial Flux_A(x,t)}{\partial x}\end{aligned} \tag{B5}$$

Changes in attractant levels in a particular box i in the discretised spatial region are then be described by:

$$\frac{dA^i(t)}{dt} = -\gamma C^i(t)A^i(t) - \frac{Flux_A^{i,i+1}(t) - Flux_A^{i-1,i}(t)}{\Delta x} \tag{B6}$$

where $Flux_A^{i-1,i}$ describes the attractant flux between boxes $i-1$ and i. Attractant fluxes across the internal box boundaries were approximated in the same way as the cell fluxes (equation (B4)), using finite differences:

$$Flux_A^{i-1,i}(t) = D_A\frac{A^i(t) - A^{i-1}(t)}{\Delta x} \tag{B7}$$

The attractant fluxes across the external boundaries of the modelled region were specified as described in Supplement B.4.

Numerical solutions of our models were obtained by numerical integration of the system of ODEs described in equations (B2, B6). Numerical integration was achieved using the R package deSolve (function ode.1D) [2].

B.2. Initial conditions

In our melanoma dataset, there were no cells in the observation region at $t = 0$. We, therefore, expect that no depletion of the attractant LPA had occurred in this region by $t = 0$, so that LPA remained at 100% of its initial concentration throughout the region at this point. Appropriate initial conditions from which to solve our models are, thus, $C(x,0) = 0$ and $A(x,0) = 1$.

In the *Dictyostelium* dataset, cells were already present in the left of the observation region at $t = 0$. The initial cell density distribution was, therefore, obtained from the cell locations at *t*=0 by first obtaining a probability density function by logspline density estimation [3–5]. This probability density function was then rescaled to ensure that the integral of $C(x,0)$ over the modelled region equalled the number of cells in the observation region at $t = 0$.

The folate in the *Dictyostelium* assay was homogeneously distributed in the gel at a concentration of 10μM prior to the addition of the cells to a folate-free trough that was cut into the gel (the edge of this trough is visible to the left of the image in Fig. 1(a)). However, there were no data on the folate distribution at the time point $t = 0$ where the first cell observations were made. Given that some cells have already moved under the gel at the left side of the region of interest at $t = 0$, it seems likely that some depletion of the folate will have occurred in this region. We, therefore, expect the folate distribution at $t = 0$ to be roughly sigmoidal in form, with low concentrations occurring near the initially folate-free trough, and a smooth increase in concentration to a maximum of 10μM occurring as we move to the right, away from the trough and the folate-depleting cells. Such a distribution of attractant at $t = 0$ can be obtained by assuming the sigmoidal functional:

$$A(x,0) = \frac{10}{1 + e^{-\delta(x-\varepsilon)}} \quad \text{(B8)}$$

The parameters *δ* and *ε* respectively describe the steepness of the increase in folate as we move to the right of the region, and the location in *x* at which half the folate is remaining. Since the precise values of these parameters were unknown, they were inferred during model fitting. We set realistic maximum and minimum values for both of these parameters (δ_{min}=0.002, δ_{max}=1, ε_{min}=0 and ε_{max}=700) by comparing the cell distribution at $t = 0$ to folate distributions obtained from equation (B8) with a range of parameter values, and selecting those values giving the realistic extremes that the attractant distribution at $t = 0$ could take (Fig. B1). There is little change to the folate distribution if *δ* is increased above the selected δ_{max}, hence the choice of this bound. Decreasing *δ* below δ_{min} causes folate to be depleted too far in advance of the cell front, or to extend too far into the initially folate-free trough area. An *ε* value of more than ε_{max} will also lead to too extensive a depleted region, while a value below ε_{min} results in high levels of folate in the trough area.

B.3. Cell Division

In both the *Dictyostelium* and melanoma datasets, the number of cells in the observation region increased substantially over time, primarily as a result of cells moving into the region across the left boundary (Figs B2A & B3A). A second contributor to increasing cell numbers is cell division. For

Dictyostelium, where the assay was run over a relatively short time interval (5.5 hours), cell division is a very minor contributor, and can reasonably be ignored. We, therefore, set the cell division rate ν of *Dictyostelium* to zero, and assumed that all increases in cell number were a result of cell movements across the left boundary (see Supplement B.4 for details). For melanoma, however, where we were interested in a time interval of 50 hours, cell division had a larger impact on the cell distribution, such that ignoring it did not give good agreement between models and data; attributing all changes in cell number to movements led to modelled cell densities that were too high at the boundary of the region. From the microscopy images, we observed that the influx of cells over the region's left boundary ceased by $t = 30$, and, since any subsequent increases in cell number can be assumed to result from cell division, we estimated $\nu = 0.004$ for melanoma by fitting an exponential curve to the data from $t = 30$ onwards (Fig B3A).

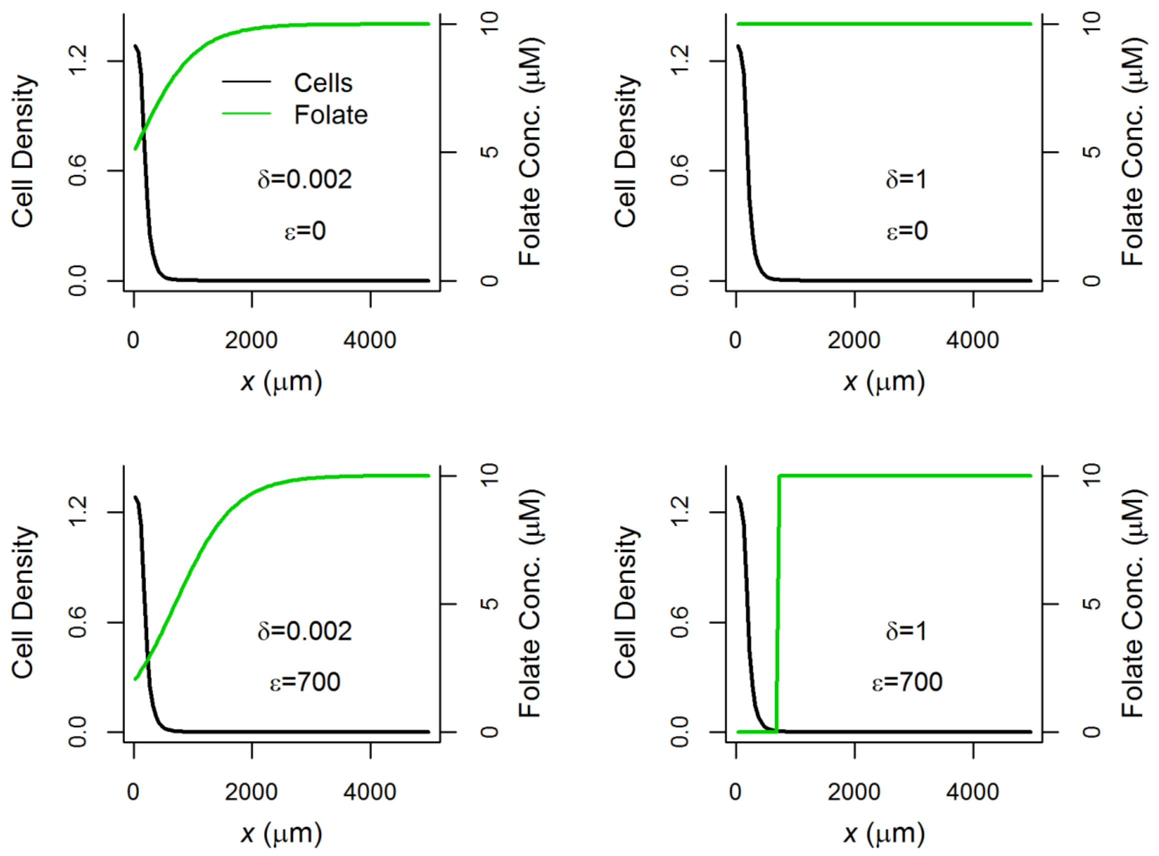

Figure B1. Extremes that the initial folate distribution was permitted to take during model fitting. Green lines show the initial attractant distributions calculated from equation (B8) using each combination of the maximum and minimum values of the parameters δ and ε. Black lines show the initial cell distribution obtained by logspline density estimation.

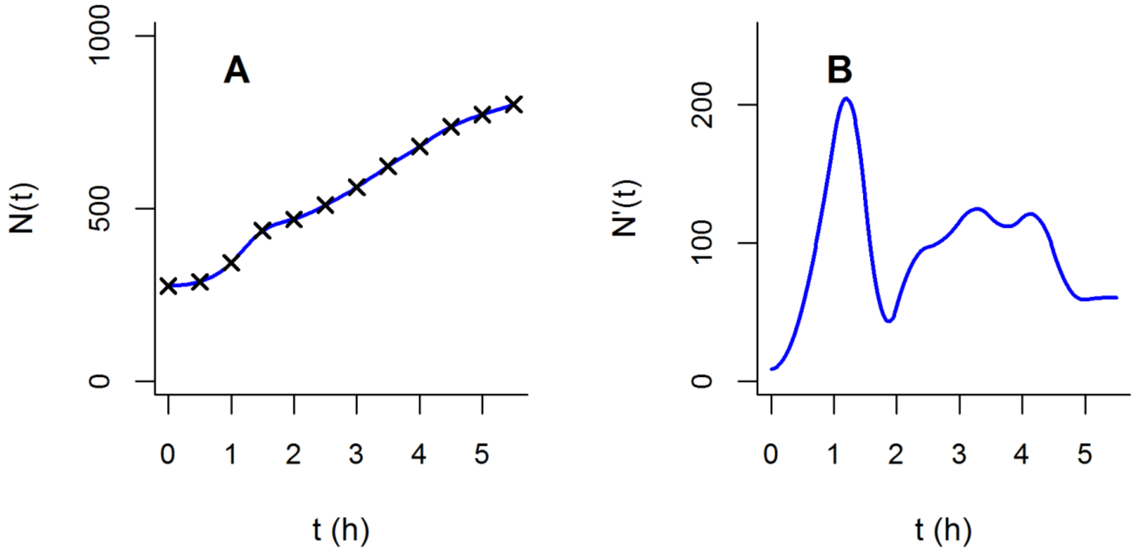

Figure B2. A) Numbers of *Dictyostelium* cells observed in microscopy images at half-hourly intervals (black points), interpolated using a cubic spline *N*(*t*) (blue line). **B)** Derivative of the spline fitted in **A**. This curve was used to define realistic boundary conditions for the cells (see Supplement B.4).

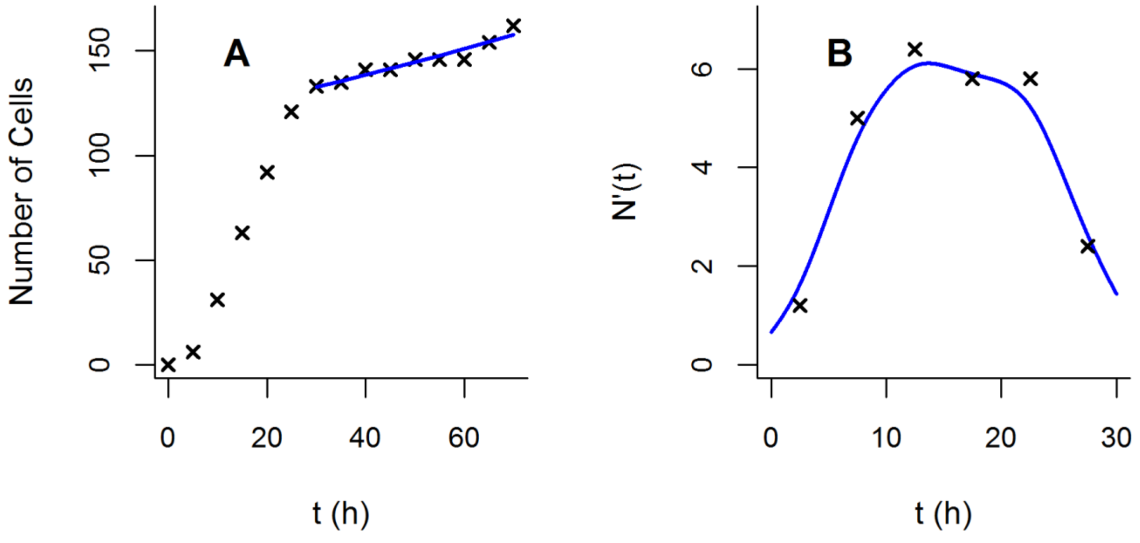

Figure B3. A) Numbers of melanoma cells observed in microscopy images at five-hourly intervals (black crosses). The blue line shows the exponential curve fitted to the data from $t = 30$ in order to estimate the rate of population growth through cell division ν. **B)** Crosses show finite difference approximations of the rate of change in cell numbers during the interval from $t = 0$ to $t = 30$, calculated from the data shown in **A.** The blue line shows the nonparametric regression curve *N'*(*t*) fitted to the points using the sm package in R [6]. This curve was used to define realistic boundary conditions for the cells (see Supplement B.4). As no new cells entered the region across the left boundary after $t = 30$, extending *N'*(*t*) beyond this point was unnecessary.

B.4. Boundary conditions

For both of our datasets it was necessary to account for movements of cells into the regions of interest across the left boundaries by incorporating appropriate boundary conditions into the models. To achieve this, we first took the time series:

$$S = \{n_j : j \in (1,\ldots,T)\} \tag{B9}$$

for each dataset, where n_j is the number of cells observed at time point $j \in (1,\ldots,T)$. We then used these data, as outlined in Figs B2-B3, to estimate smooth functions $N'(t)$ describing the rates at which the numbers of cells in the regions of interest increased over time. It can be assumed that these increases in cell numbers resulted from just two processes; movements across the region's left boundary (all cells began the assays to the left of the observation region) and cell division (see Supplement B.3). A reasonable left boundary condition would, therefore, be:

$$Flux_C^{0,1} = N'(t) - \nu \int_0^l C(x,t)\,dx \tag{B10}$$

where $Flux_C^{0,1}$ is the cell flux across the left boundary of the region. For *Dictyostelium*, given the choice of $\nu = 0$, equation (B10) reduces to:

$$Flux_C^{0,1} = N'(t) \tag{B11}$$

while, for melanoma, as no cells cross the left boundary after $t = 30$, we have:

$$Flux_C^{0,1} = \begin{cases} N'(t) - \nu \int_0^l C(x,t)\,dx & \text{if } t \in (0,30) \\ 0 & \text{if } t > 30 \end{cases} \tag{B12}$$

In both datasets, no cells crossed the right boundary during the time period considered, so we applied a zero-flux boundary condition:

$$Flux_C^{B,B+1} = 0 \tag{B13}$$

where B is the total number of boxes making up the discretised spatial region. This condition prevents any loss or gain of cell density across this boundary.

A reasonable assumption that we make for the boundary conditions for the attractants (folate and LPA) is that the flux across each region boundary equals the flux across the nearest internal box boundary in the spatial discretisation:

$$Flux_A^{0,1}(t) = Flux_A^{1,2}(t) \tag{B14}$$

$$Flux_A^{B,B+1}(t) = Flux_A^{B-1,B}(t) \tag{B15}$$

Supplement C: Weighted log-likelihood maximisation

Numerical solution of the PDE models using the method of lines (Supplement B.1) introduces error through discretisation of the models in space and time. This numerical error in the model solution leads to noise in the computation of the derivatives of the weighted log-likelihood (equation (4.4)) with respect to the parameters (via difference quotients). If the parameter difference is sufficiently large, corresponding to a low resolution representation, this numerical noise tends to average out and the weighted log-likelihood appears to be smooth (top row of Fig. C1). However, if the difference is small, corresponding to a higher resolution representation, the numerical noise does not average out and we observe spurious low-magnitude high-frequency oscillations (bottom row of Fig. C1). These numerical artefacts in the weighted log-likelihood surface cause problems for parameter inference by trapping optimisation algorithms that seek to maximise this function.

When fitting our models by the maximum weighted log-likelihood, we introduced steps to deal with the problem of numerical instabilities leading to optimisers becoming trapped on local optima. These involved first attempting to get close to the global optimum for each model by running 200 optimisations from random initial parameter sets using an optimiser (we found that the quasi-Newton BFGS algorithm performed well for our *Dictyostelium* dataset, while the Nelder-Mead algorithm was more effective at reaching high weighted log-likelihood parameter regions for the melanoma dataset). From these 200 optimisations we retained only the one that gave the highest weighted log-likelihood. One-dimensional profile weighted log-likelihood plots around these best parameter sets (using a low enough resolution for each parameter to obtain a smooth weighted log-likelihood profile) were then used to determine whether the weighted log-likelihood was actually at a maximum at the optimised value for each parameter. If the parameters had not been fully optimised, we adjusted one of the parameters that was furthest from its optimal position (selected based on the weighted log-likelihood plots) to an improved position. A re-optimisation of the full parameter set was then implemented. This process of parameter adjustment and re-optimisation was continued until re-plotting the weighted log-likelihood profiles showed that a maximum had been reached for all parameters (Fig. C2), indicating that we had reached the maximum weighted log-likelihood. Model comparison using $\mathrm{AIC_C}$ (the Akaike Information Criterion corrected for small sample sizes [7,8]) and BIC (Bayesian Information Criterion [9]) could then be carried out by calculating these statistics for each model as:

$$\mathrm{AIC_C} = -2\log\tilde{L}^* + 2k + \frac{2k(k+1)}{n-k-1} \qquad \text{(C1)}$$

$$\mathrm{BIC} = -2\log\tilde{L}^* + k\log n \qquad \text{(C2)}$$

where $\log\tilde{L}^*$ is the maximum weighted log-likelihood and k is the number of model parameters. These statistics reward models based on their fit to the data, indicated by $\log\tilde{L}^*$, and apply a complexity penalty based on k, on the assumption that all parameters are well-determined by the data.

This weighted log-likelihood maximisation procedure is very effective for obtaining a reliable estimate of the optimal parameters. However, the reliance of this method on visual inspections of the profile weighted log-likelihood and manual parameter adjustments make it labour intensive. In addition, this method does not produce an estimate of the posterior distribution of the parameters, making it difficult to assess parameter uncertainty, and restricting our access to more advanced model comparison statistics like WAIC [10]. For these reasons, we only relied on model inference using the maximum weighted log-likelihood during selection of the degrees of the polynomial functions describing the time-varying parameters (Tables S1-S2), and when determining the relative importance of the time-variance in each parameter in the best model for each dataset (Tables S5-S6). When carrying out the more important task of comparing the full set of candidate models for each dataset, we applied the inference scheme described in section 5 of the main text, which involved the development of a pseudo-posterior through multiple optimisations on bootstrap samples of the data, and thus allowed the calculation of WAIC. While this bootstrapping method allows a more advanced model comparison, it does incur high computational costs, which is why, in the face of limited cluster resources with which to parallelise this procedure, we resorted to the computationally cheaper weighted log-likelihood maximisation for the more minor model comparisons. While we note that WAIC should be preferred as the more reliable statistic, we did also compare the full set of models using AICc and BIC to check for consistency between these statistics (Tables S3-S4).

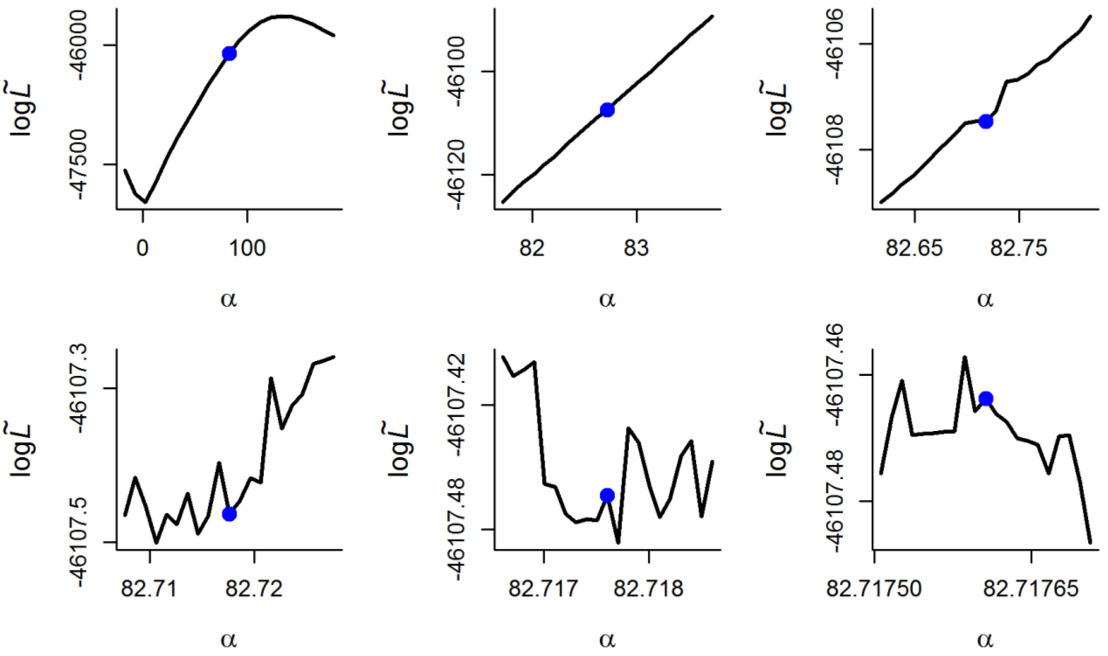

Fig. C1. One-dimensional plots of the weighted log-likelihood (equation (4.4)) against a parameter α at different resolutions. The value to which the parameter was optimised on one run of the quasi-Newton BFGS optimisation algorithm is marked with a point. Note that the optimiser has failed to reach the maximum likelihood value, and become trapped on a local optimum instead. These local optima are artefacts of the numerical noise inherent in the discretisation of the PDEs, and only appear at high resolution (i.e. when making small changes in the parameter values).

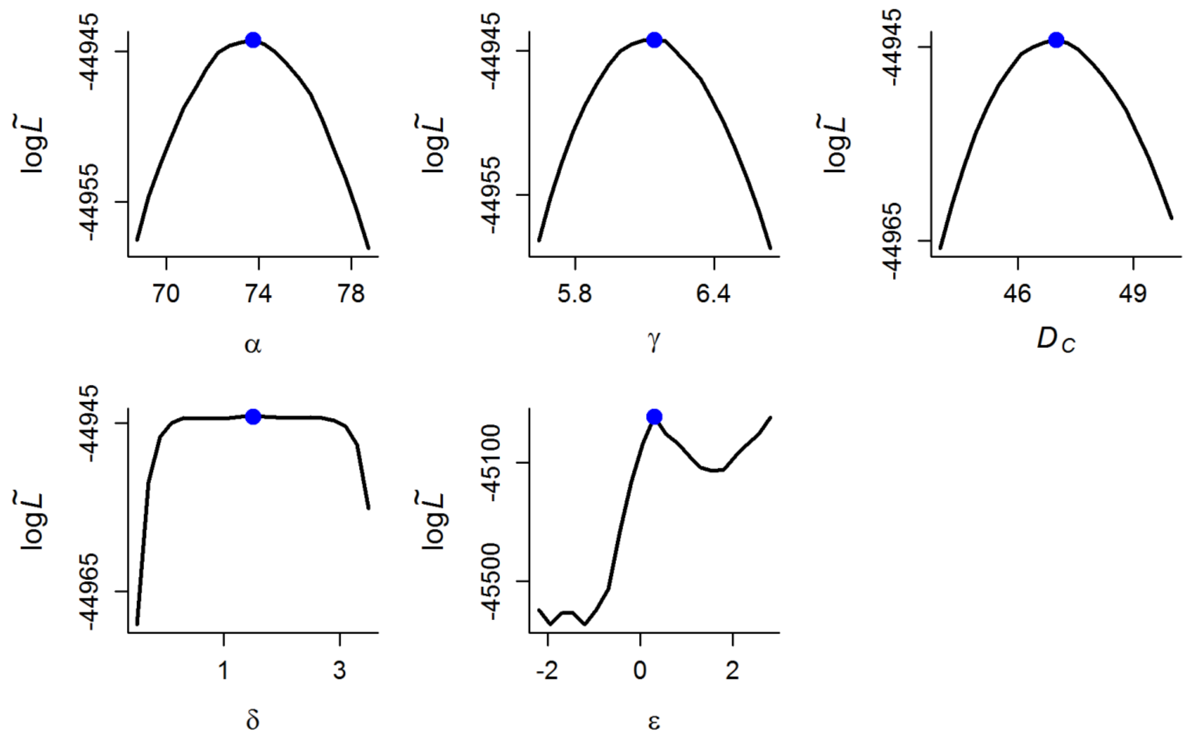

Fig. C2. One-dimensional plots of the weighted log-likelihood (equation (4.4)) landscape around the parameters for one of our models following sufficient optimisation. For each parameter, the resolution was selected to be low enough to give a relatively smooth likelihood surface. Note that all parameters have now been optimised to a true peak in the likelihood surface (compare with Fig. C1).

Supplement D: Eliminating bimodality in the pseudo-posterior

Our inference method involving multiple optimisations on many bootstrap samples of the data (see see section 5 of the main text) resulted in the production of a pseudo-posterior for each model. For both datasets, we observed bimodality in the pseudo-posteriors for all models except the simple diffusion model (Figs D1-D2). This bimodality is a result of the presence of local optima, which cause some optimisations to become trapped before they reach the maximum likelihood parameters. For both datasets, the positions of the lower-likelihood peaks in the posteriors of the more complex models roughly correspond to the position of the single likelihood peak that occurs for the diffusion model. This suggests that these lower-likelihood peaks are made up of optimisations that failed to properly fit the parameters describing the self-generated attractant gradient mechanism; a suggestion that is backed up by the fact that model outputs obtained by sampling from these lower peaks closely resembled those obtained from a diffusion-only scenario (shown in Figs S1-S2). The presence of these low-likelihood peaks in the pseudo-posteriors will affect the values of model comparison statistics calculated from these pseudo-posteriors, potentially influencing model

rankings. We, therefore, chose to isolate and use only the highest-likelihood peak when evaluating our models. This was achieved for each dataset by introducing a cut-off value in the log-likelihood for all the models except the diffusion model, which was positioned in the trough between the two peaks. Any optimisations that achieved a log-likelihood that was lower than this cut-off were discarded, and only the remaining optimisations (indicated by the blue shaded areas in Figs D1-D2) were used in the calculation of model comparison statistics (see section 5 of the main text).

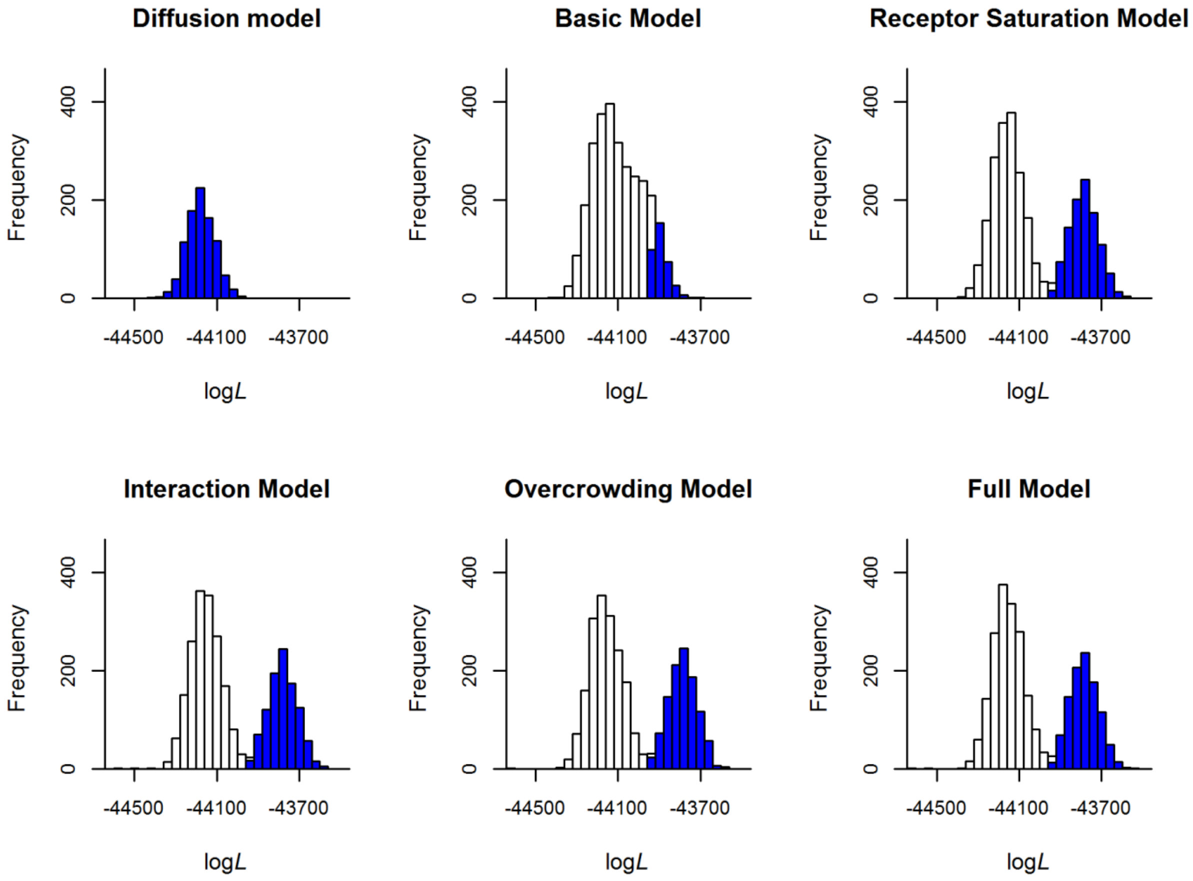

Figure D1. Histograms showing the pseudo-posteriors resulting from multiple optimisations of each model on bootstrap samples of the *Dictyostelium* data. Note that all pseudo-posteriors except that for the diffusion model exhibit bimodality (though the two peaks are fused in the case of the basic model). For all models except the diffusion model, we introduced a cut-off of $\log L = -43940$ to isolate the upper peak in the likelihood. The blue shaded areas illustrate the shapes of the pseudo-posteriors after imposing this cut-off.

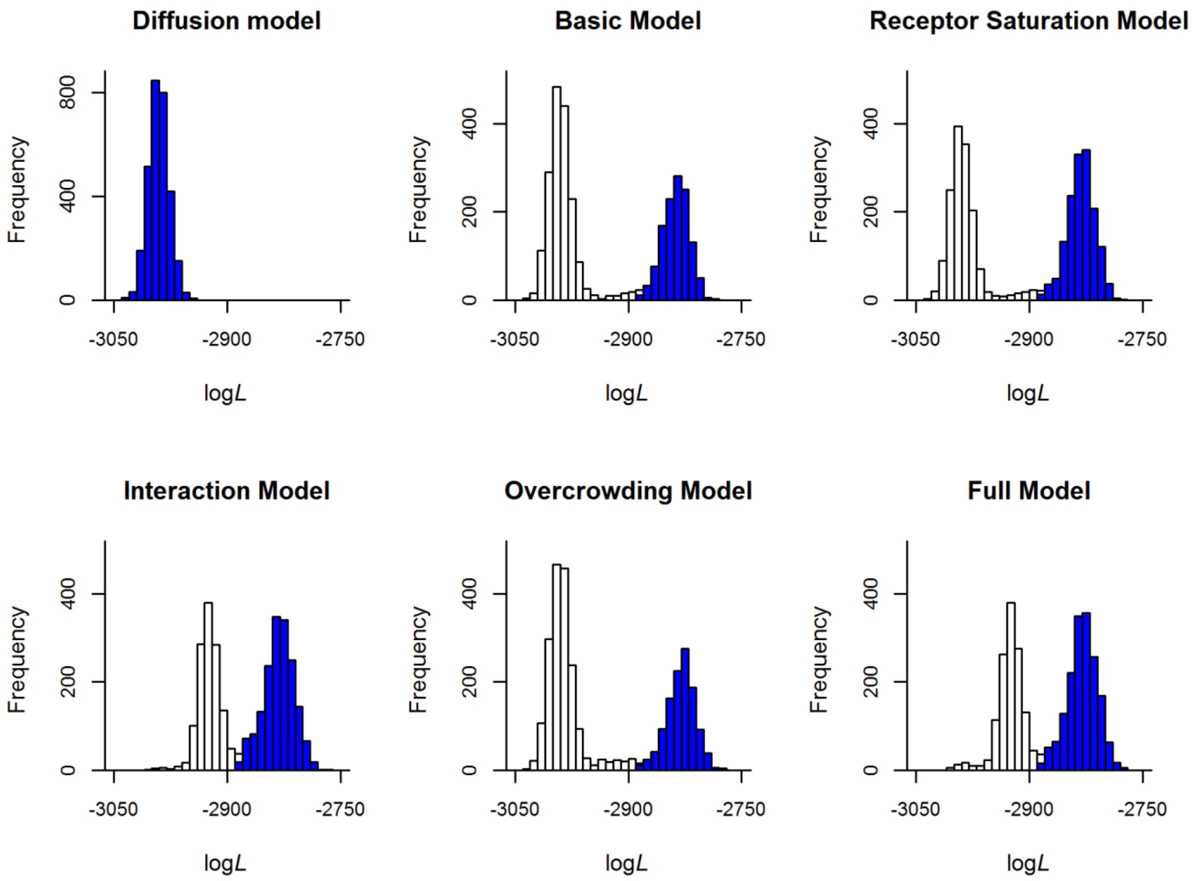

Figure D2. Histograms showing the pseudo-posteriors resulting from multiple optimisations of each model on bootstrap samples of the melanoma data. Note that all pseudo-posteriors except that for the diffusion model exhibit bimodality. For all models except the diffusion model, we introduced a cut-off of $\log L = -2885$ to isolate the upper peak in the likelihood. The blue shaded areas illustrate the shapes of the pseudo-posteriors after imposing this cut-off.

Supplement E: Comparison of alternative methods of calculating DIC and WAIC

We compared our models using WAIC values calculated using a pseudo-posterior obtained by fitting the models to many bootstrap datasets (see main text, section 5). To verify whether this method produces results comparable to sampling from a true posterior, we carried out an additional study using the radiocarbon dataset from the sm() package in R [6], which describes the radiocarbon age of Irish oak in comparison to its true calendar age. This involved comparing the fits of polynomial models of degrees one to nine (Fig. E1) using DIC (Deviance Information Criterion [11]) and WAIC values calculated from the true posterior and from the pseudo-posterior obtained by our bootstrapping method. Note that we have not compared our models based in DIC in the main text, since we encountered issues with negative values being estimated for the effective number of

parameters (a known issue with this comparison statistic), rendering DIC less reliable than the more recently developed WAIC.

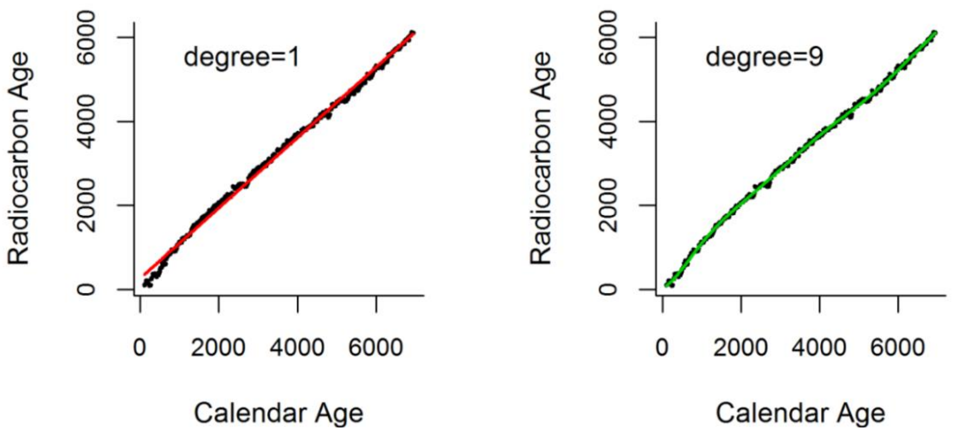

Fig. E1. Fits of polynomials of degrees one and nine to the radiocarbon dataset

Calculation of DIC and WAIC from the true posterior

The polynomial models fitted to the data take the form:

$$\mathbf{y} = \mathbf{B}\boldsymbol{\beta} + \boldsymbol{\varepsilon} \tag{E1}$$

where $\mathbf{y} = (y_1, ..., y_n)^T$ is the vector of radiocarbon age observations (*n*=343), $\boldsymbol{\beta} = (\beta_1, ..., \beta_k)^T$ is the vector of coefficients (*k* is equal to the degree of the polynomial minus one) and $\boldsymbol{\varepsilon} = (\varepsilon_1, ..., \varepsilon_n)^T$ is iid (independent and identically distributed) Gaussian error, with mean zero and variance σ^2. For each model considered, σ^2 was estimated by fitting to the data and calculating the variance of the residuals. The design matrix $\mathbf{B}$ is given by:

$$\mathbf{B} = \begin{pmatrix} x_1^{\ 0} & \cdots & x_1^{\ (k-1)} \\ \vdots & \ddots & \vdots \\ x_n^{\ 0} & \cdots & x_n^{\ (k-1)} \end{pmatrix} \tag{E2}$$

where $\mathbf{x} = \left(x_1, \ldots, x_n\right)^T$ is the calendar age covariate.

Gaussian priors with mean zero and variance ζ^2 were applied to each of the parameters. We specified vague prior distributions where $\zeta^2 = 1\times10^6$. The likelihood is given by:

$$P\left(\mathbf{y} \mid \mathbf{x}, \boldsymbol{\beta}, \sigma^2\right) = \left(2\pi\sigma^2\right)^{-\frac{n}{2}} \exp\left\{\frac{-(\mathbf{y}-\mathbf{B}\boldsymbol{\beta})^T(\mathbf{y}-\mathbf{B}\boldsymbol{\beta})}{2\sigma^2}\right\} \tag{E3}$$

As the priors and likelihood are Gaussian distributions, the posterior is Gaussian also, and is given by:

$$P\left(\boldsymbol{\beta} \middle| \mathbf{x}, \mathbf{y}, \sigma^2, \zeta^2\right) = N(\boldsymbol{\mu}, \boldsymbol{\Sigma}) \tag{E4}$$

where

$$\boldsymbol{\mu} = \left(\mathbf{B}^T\mathbf{B} + \frac{\sigma^2}{\zeta^2}\mathbf{I}\right)^{-1} \mathbf{B}^T\mathbf{y} \tag{E5}$$

$$\boldsymbol{\Sigma} = \sigma^2\left(\mathbf{B}^T\mathbf{B} + \frac{\sigma^2}{\zeta^2}\mathbf{I}\right)^{-1} \tag{E6}$$

We drew a sample of *m*=20,000 parameter sets $(\boldsymbol{\beta}_1, \ldots, \boldsymbol{\beta}_m)$ from the posterior distribution for each model and, using the likelihood function stated above (equation (E3)), calculated the DIC as:

$$\mathrm{DIC} = \frac{2\sum_{i=1}^{m} -2\log\left\{P\left(\mathbf{y} \mid \mathbf{x}, \boldsymbol{\beta}_i, \sigma^2\right)\right\}}{m} + 2\log\left\{P\left(\mathbf{y} \mid \mathbf{x}, \bar{\boldsymbol{\beta}}, \sigma^2\right)\right\} \tag{E7}$$

where $\bar{\beta}$ are the mean values of the parameters, and the WAIC as:

$$\begin{aligned} \mathrm{WAIC} = & -2\sum_{j=1}^{n}\log\left\{\frac{1}{m}\sum_{i=1}^{m}P\left(y_j \mid x_j, \boldsymbol{\beta}_i, \sigma^2\right)\right\} \\ & +2\sum_{j=1}^{n}\left\{\frac{1}{m}\left(\sum_{i=1}^{m}\left[\log\left\{P\left(y_j \mid x_j, \boldsymbol{\beta}_i, \sigma^2\right)\right\}\right]^2\right) - \left[\frac{1}{m}\sum_{i=1}^{m}\log\left\{P\left(y_j \mid x_j, \boldsymbol{\beta}_i, \sigma^2\right)\right\}\right]^2\right\} \end{aligned} \tag{E8}$$

Calculation of DIC and WAIC from bootstrap samples

The data were sampled with replacement to generate *m*=20,000 bootstrap datasets of the same dimension *n* as the original dataset, each consisting of a vector of radiocarbon age observations $\mathbf{r}_i = (r_{i,1}, \ldots, r_{i,n})^T$ and the associated calendar ages $\mathbf{q}_i = (q_{i,1}, \ldots, q_{i,n})^T$, where $i \in (1, \ldots, m)$. Since we chose a vague prior for the regression parameters (i.e. with a large value of the variance hyperparameter ζ^2 in equations E4-E6), maximum likelihood parameter estimates will be effectively the same as maximum a posteriori estimates. We, therefore, fitted the nine polynomial models to each of the bootstrap datasets using maximum likelihood to obtain a sample of parameter sets $(\boldsymbol{\theta}_1, \ldots, \boldsymbol{\theta}_m)$ that we take as an approximation of a posterior distribution. This

‘pseudo-posterior’ can be used to estimate the DIC and WAIC in two alternative ways. The first uses the parameter sets obtained from the bootstrap data (and their mean $\bar{\theta}$), with only the true un-bootstrapped data as follows:

$$\mathrm{DIC_A} = \frac{2\sum_{i=1}^{m} -2\log\left\{P\left(\mathbf{y}\mid\mathbf{x},\boldsymbol{\theta}_i,\sigma^2\right)\right\}}{m} + 2\log\left\{P\left(\mathbf{y}\mid\mathbf{x},\bar{\boldsymbol{\theta}},\sigma^2\right)\right\} \quad \text{(E9)}$$

$$\begin{aligned}\mathrm{WAIC_A} &= -2\sum_{j=1}^{n}\log\left\{\frac{1}{m}\sum_{i=1}^{m}P\left(y_j\mid x_j,\boldsymbol{\theta}_i,\sigma^2\right)\right\} \\ &+2\sum_{j=1}^{n}\left\{\frac{1}{m}\left(\sum_{i=1}^{m}\left[\log\left\{P\left(y_j\mid x_j,\boldsymbol{\theta}_i,\sigma^2\right)\right\}\right]^2\right)-\left[\frac{1}{m}\sum_{i=1}^{m}\log\left\{P\left(y_j\mid x_j,\boldsymbol{\theta}_i,\sigma^2\right)\right\}\right]^2\right\}\end{aligned} \quad \text{(E10)}$$

In the second method we incorporate the bootstrap data into the calculations:

$$\mathrm{DIC_B} = \frac{2\sum_{i=1}^{m} -2\log\left\{P\left(\mathbf{r}_i\mid\mathbf{q}_i,\boldsymbol{\theta}_i,\sigma^2\right)\right\}}{m} + 2\log\left\{P\left(\mathbf{y}\mid\mathbf{x},\bar{\boldsymbol{\theta}},\sigma^2\right)\right\} \quad \text{(E11)}$$

$$\begin{aligned}\mathrm{WAIC_B} &= -2\sum_{j=1}^{n}\log\left\{\frac{1}{m}\sum_{i=1}^{m}P\left(r_{i,j}\mid q_{i,j},\boldsymbol{\theta}_i,\sigma^2\right)\right\} \\ &+2\sum_{j=1}^{n}\left\{\frac{1}{m}\left(\sum_{i=1}^{m}\left[\log\left\{P\left(r_{i,j}\mid q_{i,j},\boldsymbol{\theta}_{i,i},\sigma^2\right)\right\}\right]^2\right)-\left[\frac{1}{m}\sum_{i=1}^{m}\log\left\{P\left(r_{i,j}\mid q_{i,j},\boldsymbol{\theta}_{i,i},\sigma^2\right)\right\}\right]^2\right\}\end{aligned} \quad \text{(E12)}$$

Results

We found a generally strong correspondence between the standard DIC and WAIC values and our approximations (Fig. E2), with correlation coefficients in excess of 0.999 for all relationships, except that between the standard WAIC and $\mathrm{WAIC_B}$, which had a correlation coefficient of 0.98. The standard DIC and WAIC both select the eighth degree polynomial from the nine candidate models (Table E1). $\mathrm{DIC_A}$ and $\mathrm{WAIC_A}$ are successful in selecting this same best model, and in exactly replicating the full model ranking observed for the standard DIC and WAIC, suggesting that these statistics are a valid approximation of the standard DIC and WAIC. $\mathrm{DIC_B}$ and $\mathrm{WAIC_B}$ were less successful, showing a preference for the ninth degree polynomial. Based on these results, we chose to use the $\mathrm{WAIC_A}$ approximation when comparing our models of cell movement.

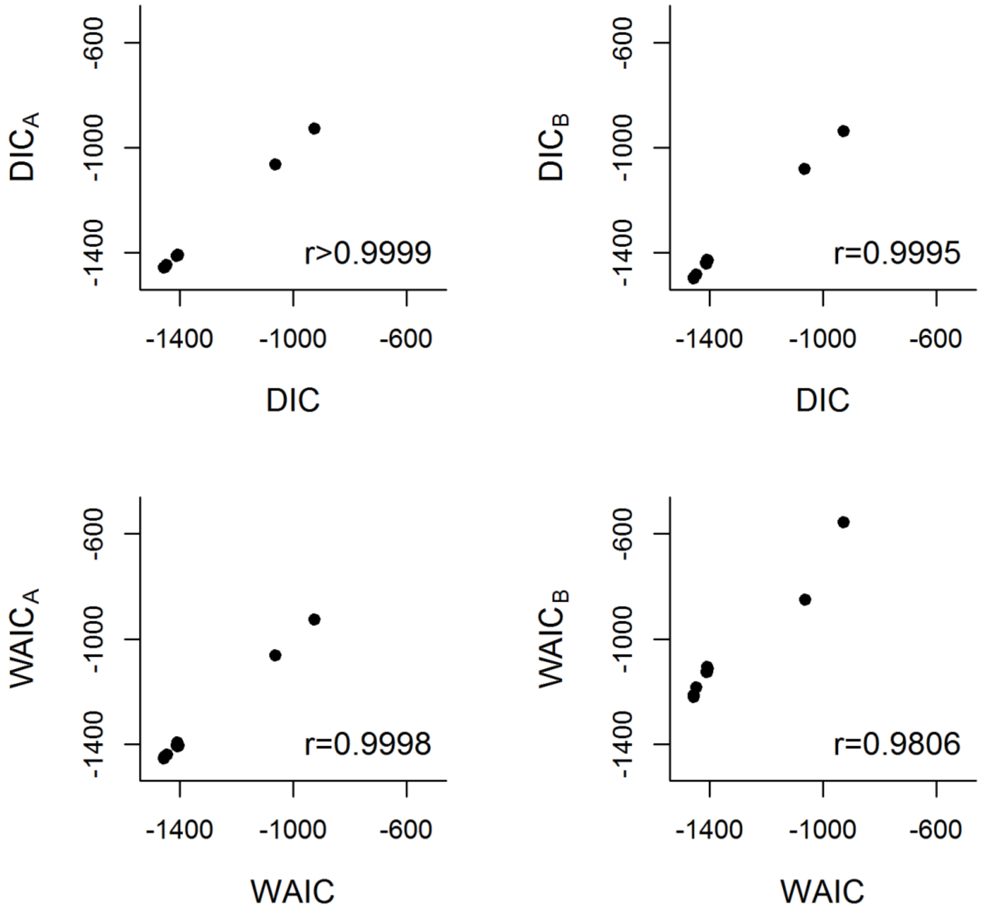

Fig. E2. Plots of the DIC and WAIC approximations obtained for the nine polynomial models through bootstrapping against the standard DIC and WAIC values obtained for the models by direct sampling from the posterior. The value of Pearson's correlation coefficient is indicated for each comparison.

Table E1. DIC and WAIC values for each polynomial model fitted to the radiocarbon data (Fig. E1). The standard values of each statistic are calculated using the true posterior, while the alternative estimates are obtained through our bootstrapping technique. The best model based on each statistic is indicated *.

Polynomial Degree	Standard DIC	DIC$_A$	DIC$_B$	Standard WAIC	WAIC$_A$	WAIC$_B$
1	-927.31	-926.39	-936.30	-926.79	-924.56	-555.77
2	-1065.26	-1063.28	-1079.56	-1064.25	-1060.74	-850.45
3	-1408.59	-1407.97	-1424.63	-1408.22	-1406.50	-1106.11
4	-1407.05	-1406.37	-1427.20	-1406.58	-1404.03	-1112.05
5	-1412.40	-1410.19	-1437.82	-1411.23	-1403.77	-1124.85
6	-1411.10	-1407.39	-1441.23	-1409.49	-1393.46	-1123.97
7	-1448.16	-1445.32	-1481.31	-1447.07	-1439.37	-1183.88
8	-1457.18*	-1455.92*	-1492.69	-1456.81*	-1452.47*	-1212.81
9	-1456.01	-1453.91	-1495.43*	-1455.65	-1446.31	-1219.04*

Supplement F: Calculation of standard errors for WAIC

The WAIC was calculated for our cell movement models as:

$$\begin{aligned}\text{WAIC} &= -2\sum_{j=1}^{n}\log\left\{\frac{1}{m}\sum_{i=1}^{m}P\left(y_j\,|\,\boldsymbol{\theta}_i\right)\right\}\\ &+2\sum_{j=1}^{n}\left\{\frac{1}{m}\left(\sum_{i=1}^{m}\left[\log\left\{P\left(y_j\,|\,\boldsymbol{\theta}_i\right)\right\}\right]^2\right)-\left[\frac{1}{m}\sum_{i=1}^{m}\log\left\{P\left(y_j\,|\,\boldsymbol{\theta}_i\right)\right\}\right]^2\right\}\end{aligned} \quad \text{(F1)}$$

To calculate the variance of the first term, we first obtained the variances of the mean likelihoods of each observation y_j using:

$$\begin{aligned}\text{var}\left(\frac{1}{m}\sum_{i=1}^{m}P\left(y_j\,|\,\boldsymbol{\theta}_i\right)\right) &= \frac{1}{m}\text{var}\left(P\left(y_j\,|\,\boldsymbol{\theta}\right)\right)\\ &= \frac{1}{m}\left(\frac{1}{m}\sum_{i=1}^{m}\left[\left\{P\left(y_j\,|\,\boldsymbol{\theta}_i\right)-\frac{1}{m}\sum_{i=1}^{m}P\left(y_j\,|\,\boldsymbol{\theta}_i\right)\right\}^2\right]\right)\end{aligned} \quad \text{(F2)}$$

The univariate delta method was then applied to get the variances of the log mean likelihoods as:

$$\text{var}\left(\log\left\{\frac{1}{m}\sum_{i=1}^{m}P\left(y_j\,|\,\boldsymbol{\theta}_i\right)\right\}\right)=\left(\frac{1}{\frac{1}{m}\sum_{i=1}^{m}P\left(y_j\,|\,\boldsymbol{\theta}_i\right)}\right)^2\text{var}\left(\frac{1}{m}\sum_{i=1}^{m}P\left(y_j\,|\,\boldsymbol{\theta}_i\right)\right) \quad \text{(F3)}$$

and the variance of the sum of the log mean likelihoods was obtained as:

$$\text{var}\left(\sum_{j=1}^{n}\log\left\{\frac{1}{m}\sum_{i=1}^{m}P\left(y_j\,|\,\boldsymbol{\theta}_i\right)\right\}\right)=\sum_{j=1}^{n}\text{var}\left(\log\left\{\frac{1}{m}\sum_{i=1}^{m}P\left(y_j\,|\,\boldsymbol{\theta}_i\right)\right\}\right) \quad \text{(F4)}$$

The variance of the second term in the WAIC was obtained by first calculating the variance of the sample variance of the log likelihood of each observation y_j as:

$$\text{var}\left\{\frac{1}{m}\left(\sum_{i=1}^{m}\left[\log\left\{P\left(y_j\,|\,\boldsymbol{\theta}_i\right)\right\}\right]^2\right)-\left[\frac{1}{m}\sum_{i=1}^{m}\log\left\{P\left(y_j\,|\,\boldsymbol{\theta}_i\right)\right\}\right]^2\right\}=\frac{\left(m-1\right)^2}{m^3}\mu_4-\frac{\left(m-1\right)\left(m-3\right)\mu_2^2}{m^3} \quad \text{(F5)}$$

where μ_2 and μ_4 are the 2nd and 4th central moments of $\log\left\{P\left(y_j\,|\,\boldsymbol{\theta}_i\right)\right\}$, calculated by:

$$\mu_2 = \frac{1}{m}\sum_{i=1}^{m}\left(\left[\log\left\{P\left(y_j \mid \boldsymbol{\theta}_i\right)\right\} - \frac{1}{m}\sum_{i=1}^{m}\log\left\{P\left(y_j \mid \boldsymbol{\theta}_i\right)\right\}\right]^2\right)$$
$$\mu_4 = \frac{1}{m}\sum_{i=1}^{m}\left(\left[\log\left\{P\left(y_j \mid \boldsymbol{\theta}_i\right)\right\} - \frac{1}{m}\sum_{i=1}^{m}\log\left\{P\left(y_j \mid \boldsymbol{\theta}_i\right)\right\}\right]^4\right) \tag{F6}$$

We then sum these variances of sample variances to get:

$$\operatorname{var}\left[\sum_{j=1}^{n}\left\{\frac{1}{m}\left(\sum_{i=1}^{m}\left[\log\left\{P\left(y_j \mid \boldsymbol{\theta}_i\right)\right\}\right]^2\right) - \left[\frac{1}{m}\sum_{i=1}^{m}\log\left\{P\left(y_j \mid \boldsymbol{\theta}_i\right)\right\}\right]^2\right\}\right]$$
$$= \sum_{j=1}^{n}\operatorname{var}\left\{\frac{1}{m}\left(\sum_{i=1}^{m}\left[\log\left\{P\left(y_j \mid \boldsymbol{\theta}_i\right)\right\}\right]^2\right) - \left[\frac{1}{m}\sum_{i=1}^{m}\log\left\{P\left(y_j \mid \boldsymbol{\theta}_i\right)\right\}\right]^2\right\} \tag{F7}$$

The standard error of the full WAIC can be obtained as:

$$\operatorname{se}\left(\mathrm{WAIC}\right) = 2\sqrt{\operatorname{var}\left(\sum_{j=1}^{n}\log\left\{\frac{1}{m}\sum_{i=1}^{m}P\left(y_j \mid \boldsymbol{\theta}_i\right)\right\}\right) + \operatorname{var}\left[\sum_{j=1}^{n}\left\{\frac{1}{m}\left(\sum_{i=1}^{m}\left[\log\left\{P\left(y_j \mid \boldsymbol{\theta}_i\right)\right\}\right]^2\right) - \left[\frac{1}{m}\sum_{i=1}^{m}\log\left\{P\left(y_j \mid \boldsymbol{\theta}_i\right)\right\}\right]^2\right\}\right]} \tag{F8}$$

Supplementary Figures

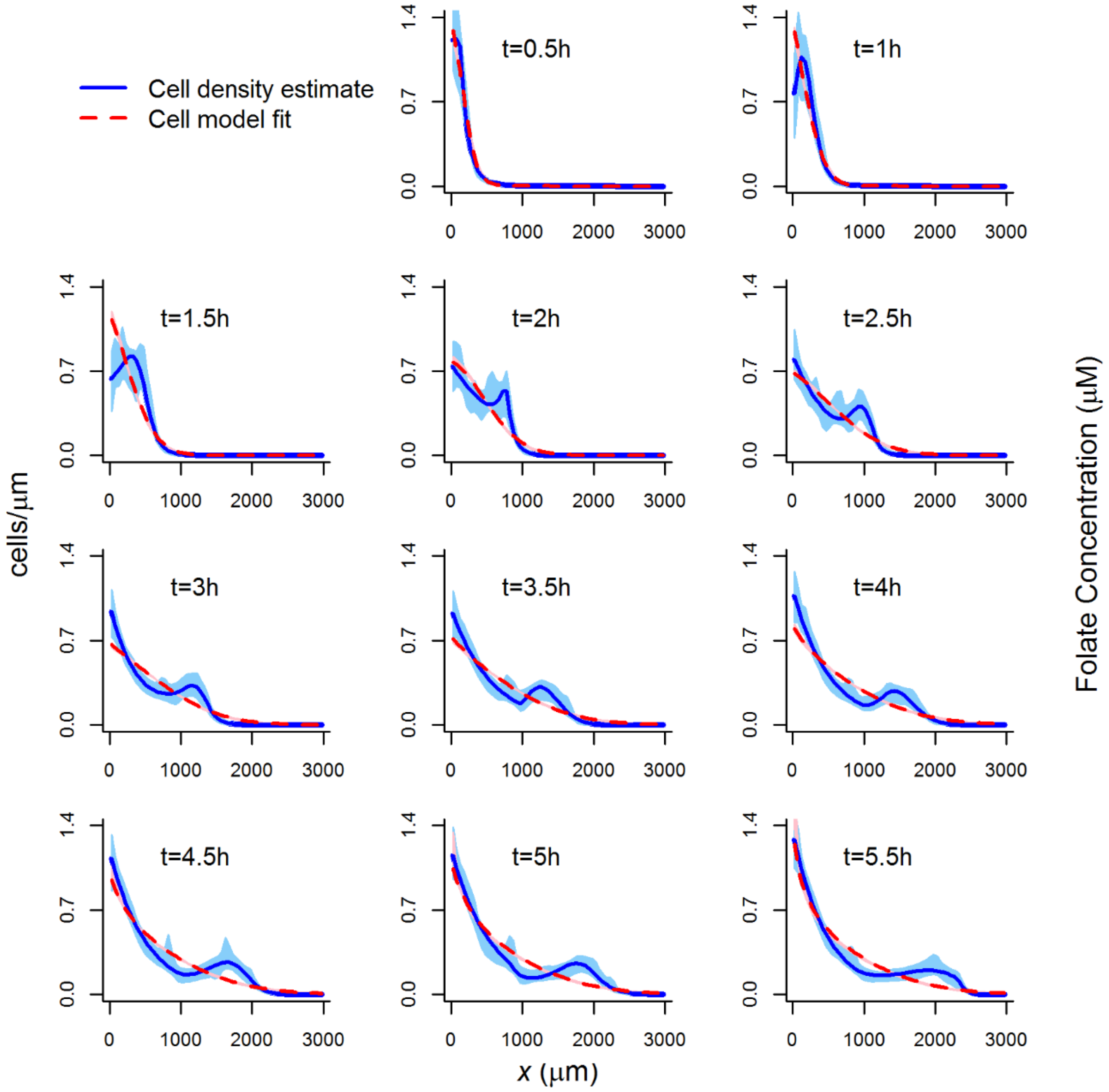

Figure S1. Dashed red lines show *Dictyostelium* cell distributions at half-hour intervals produced by the diffusion model (equation (3.2)) using the optimised parameters from the bootstrap optimisation that gave the highest value of the weighted log-likelihood (equation (4.4)). Pink shaded areas show the 95 percentile interval for the modelled cell densities, based on 200 samples from the pseudo-posterior. The corresponding LPA distributions predicted by this model are indicated by green dotted lines. Cell distributions obtained from the data using logspline density estimation [3–5] are shown by blue lines, with 95 percentile intervals obtained using 10,000 bootstrap samples of the data indicated by blue shaded areas.

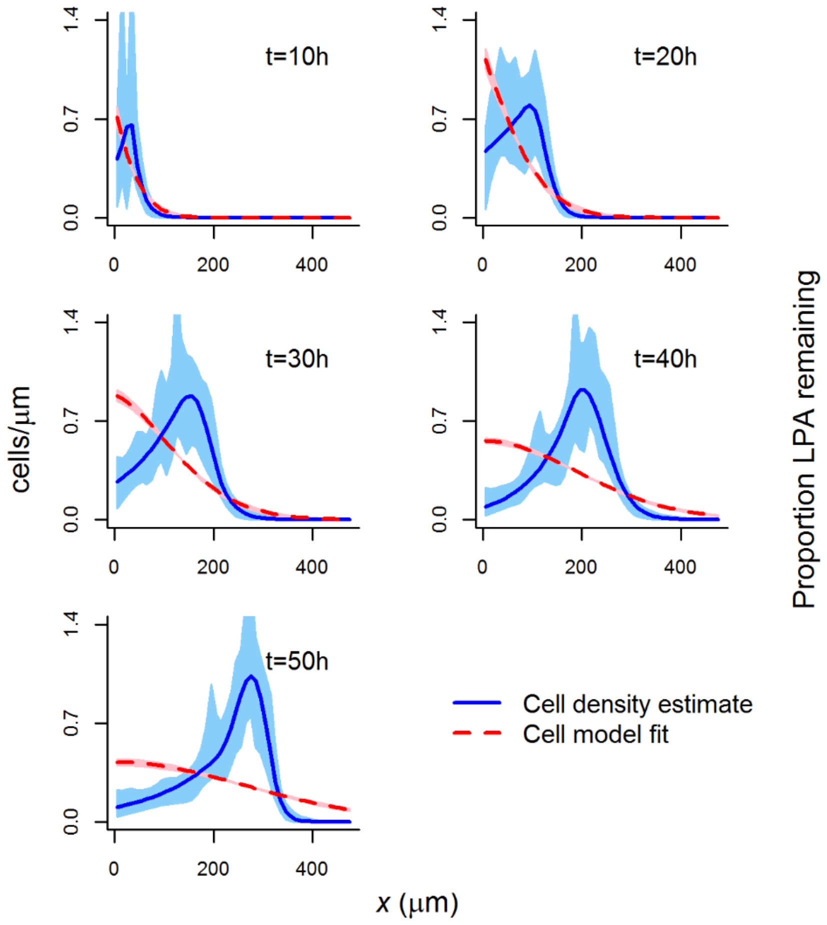

Figure S2. Dashed red lines show melanoma cell distributions at half-hour intervals produced by the diffusion model (equation (3.2)) using the optimised parameters from the bootstrap optimisation that gave the highest value of the weighted log-likelihood (equation (4.4)). Pink shaded areas show the 95 percentile interval for the modelled cell densities, based on 200 samples from the pseudo-posterior. The corresponding LPA distributions predicted by this model are indicated by green dotted lines. Cell distributions obtained from the data using logspline density estimation [3–5] are shown by blue lines, with 95 percentile intervals obtained using 10,000 bootstrap samples of the data indicated by blue shaded areas.

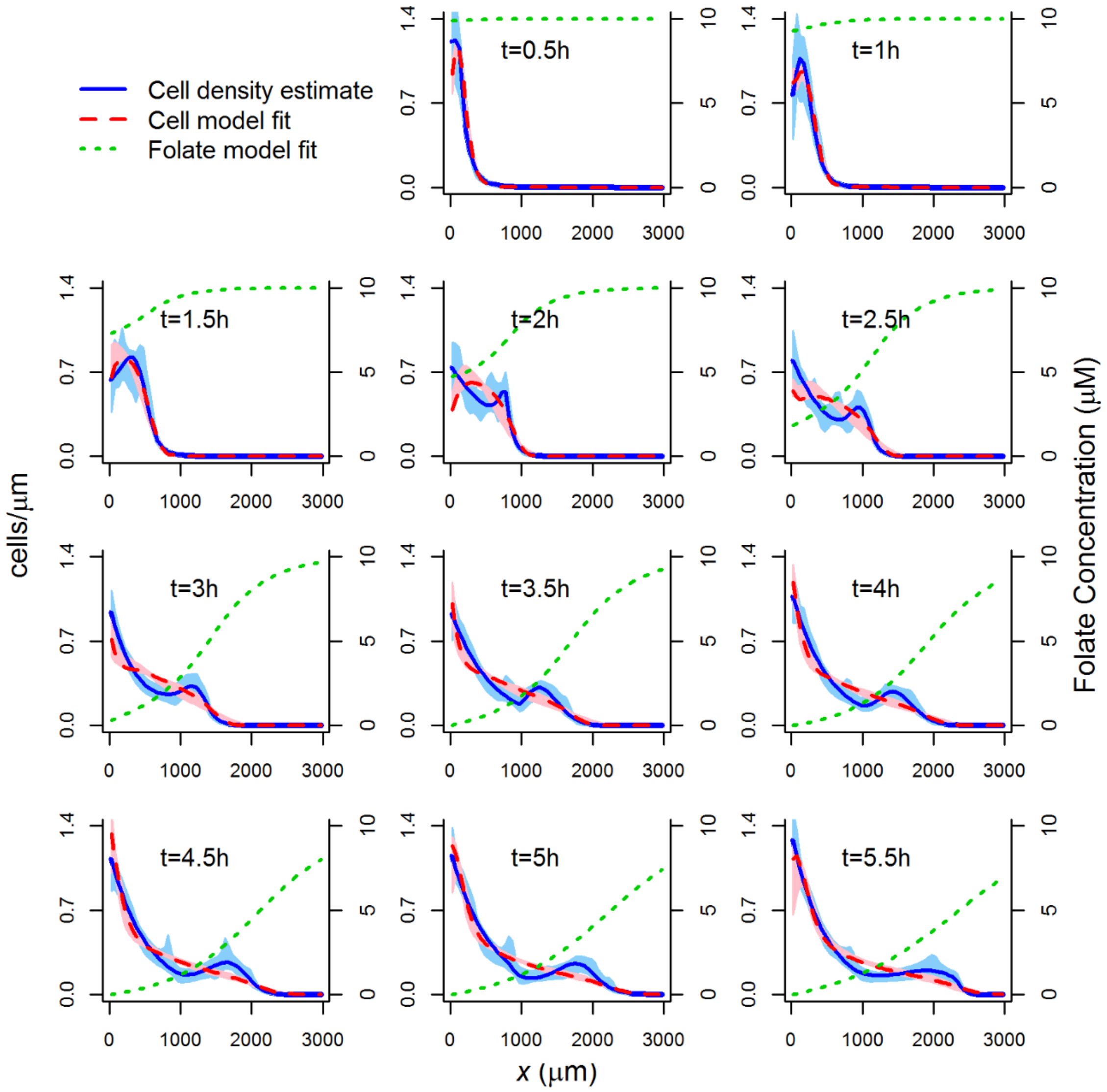

Figure S3. Dashed red lines show *Dictyostelium* cell distributions at half-hour intervals produced by the basic model (equation (3.3)) using the optimised parameters from the bootstrap optimisation that gave the highest value of the weighted log-likelihood (equation (4.4)). Pink shaded areas show the 95 percentile interval for the modelled cell densities, based on 200 samples from the pseudo-posterior. The corresponding LPA distributions predicted by this model are indicated by green dotted lines. Cell distributions obtained from the data using logspline density estimation [3,5,4] are shown by blue lines, with 95 percentile intervals obtained using 10,000 bootstrap samples of the data indicated by blue shaded areas.

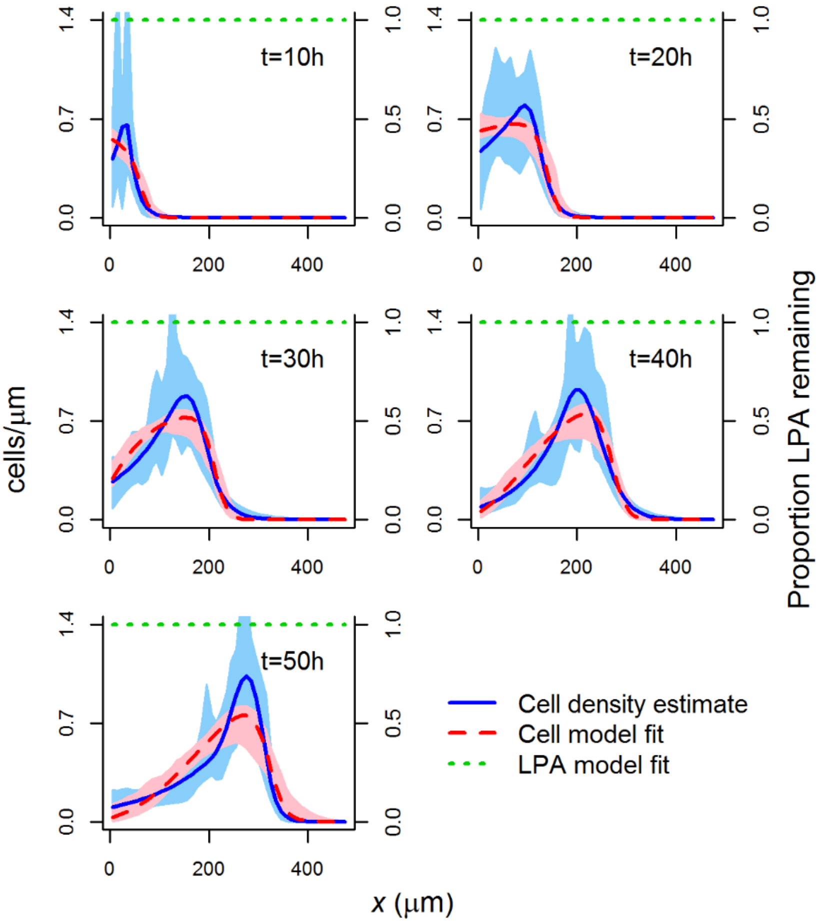

Figure S4. Dashed red lines show melanoma cell distributions at half-hour intervals produced by the basic model (equation (3.3)) using the optimised parameters from the bootstrap optimisation that gave the highest value of the weighted log-likelihood (equation (4.4)). Pink shaded areas show the 95 percentile interval for the modelled cell densities, based on 200 samples from the pseudo-posterior. The corresponding LPA distributions predicted by this model are indicated by green dotted lines. Cell distributions obtained from the data using logspline density estimation [3,5,4] are shown by blue lines, with 95 percentile intervals obtained using 10,000 bootstrap samples of the data indicated by blue shaded areas.

Supplementary Tables

Table S1: Selection of the degree of the polynomials used to describe the time-varying parameters for the *Dictyostelium* dataset, based on fits of the full model (equation (3.8)) with different degrees. Both AICc and BIC show a clear preference for a degree of three. Based on these results, we use a polynomial degree of three when fitting the remaining models to this dataset (see Table 1 in the main text).

**Degree**	**log$\tilde{L}^*$**	**AICc**	**BIC**
0	-44114.5	88249.0	88316.3
1	-43929.2	87886.5	87980.8
2	-43792.8	87621.7	87742.8
3	-43771.0	87586.1*	87734.1*
4	-43771.0	87594.1	87769.0

Table S2: Selection of the degree of the polynomials used to describe the time-varying parameters for the melanoma dataset, based on fits of the full model (equation (3.8)) with different degrees. AICc shows a strong preference for a degree of one, while BIC (a comparison statistic known for its tendency to select models that are overly simple [12]) shows only a slight preference for a degree of zero (i.e. no time variance). Based on these results, we use a polynomial degree of one when fitting the remaining models to this dataset (see Table 1 in the main text).

**Degree**	**log$\tilde{L}^*$**	**AICc**	**BIC**
0	-2850.5	5717.3	5751.4*
1	-2838.5	5701.5*	5752.5
2	-2837.7	5708.5	5776.2

Table S3: Comparison of the six models fitted to the *Dictyostelium* data using AICc and BIC values calculated using the maximum weighted log-likelihood fits (Supplement C).

**Model**	**AICc**	**BIC**
Diffusion	88356.81	88383.75
Basic	87831.87	87932.83
Receptor Saturation	87587.29	87694.98*
Interaction	87584.05*	87725.36
Overcrowding	87589.00	87703.41
Full	87586.06	87734.09

Table S4: Comparison of the six models fitted to the melanoma data. AICc and BIC values were calculated using the maximum weighted log-likelihood fits (Supplement C).

Model	AICc	BIC
Diffusion	6003.3	6011.9
Basic	5711.9	5741.8
Receptor Saturation	5701.1*	5735.3*
Interaction	5702.1	5748.9
Overcrowding	5703.2	5741.5
Full	5701.5	5752.5

Table S5: Consequences of dropping the time-variance of parameters in the receptor saturation model fitted to the *Dictyostelium* dataset (the best model for this dataset based on WAIC; Table 1). Removing variation in α gives poorer (higher) values of AICc and BIC, while removing variation in D_C improves BIC but gives a poorer AICc. Making γ constant improves BIC and has little effect on AICc.

Time-varying parameters	$\log\tilde{L}^*$	AICc	BIC
α,γ,D_C	-43777.6	87587.3*	87695.0
γ,D_C	-43823.5	87673.0	87760.6
α,D_C	-43780.9	87588.0	87675.5*
α,γ	-43783.7	87593.4	87680.9
α	-43830.6	87681.3	87748.6
γ	-43853.0	87726.0	87793.4
D_C	-44094.8	88209.6	88276.9

Table S6: Consequences of dropping the time-variance of parameters in the overcrowding model fitted to the melanoma dataset (the best model for this dataset based on WAIC; Table 1). Note that there is virtually no change in the maximum weighted log-likelihood provided that α is retained as a time-varying parameter. There is also no reduction in either AICc or BIC unless both α and γ are removed as time-varying parameters, suggesting that these two parameters are able to compensate for one another to some degree.

Time-varying parameters	$\log\tilde{L}^*$	AICc	BIC
α,γ,D_C	-2842.4	5703.2	5741.5
γ,D_C	-2843.5	5703.3	5737.4
α,D_C	-2842.4	5701.2	5735.3
α,γ	-2842.4	5701.2	5735.3
α	-2842.4	5699.1*	5729.0*
γ	-2843.9	5702.0	5731.9
D_C	-2849.1	5712.3	5742.2

Supplementary References

1. Schiesser, W. E. & Griffiths, G. W. 2009 *A compendium of partial differential equation models: method of lines analysis with Matlab.* New York: Cambridge University Press.

2. Soetaert, K., Petzoldt, T. & Setzer, R. W. 2010 Solving Differential Equations in R: Package deSolve. *J. Stat. Softw.* **33**, 1–25.

3. Kooperberg, C. 2013 Logspline density estimation routines.

4. Kooperberg, C. & Stone, C. J. 1992 Logspline density estimation for censored data. *J. Comput. Graph. Stat.* **1**, 301–328. (doi:10.1016/S0167-7152(99)00097-8)

5. Stone, C. J., Hansen, M. H., Kooperberg, C. & Truong, Y. K. 1997 Polynomial splines and their tensor products in extended linear modeling: 1994 Wald memorial lecture. *Ann. Stat.* **25**, 1371–1470. (doi:10.1214/aos/1031594728)

6. Bowman, A. W. & Azzalini, A. 2014 R package 'sm': nonparametric smoothing methods.

7. Hurvich, C. M. & Tsai, C.-L. 1989 Regression and time series model selection in small samples. *Biometrika* **76**, 297–307. (doi:10.1093/biomet/76.2.297)

8. Akaike, H. 1974 A new look at the statistical model identification. *IEEE Trans. Automat. Contr.* **19**, 716–723. (doi:10.1109/TAC.1974.1100705)

9. Schwarz, G. 1978 Estimating the dimension of a model. *Ann. Stat.* **6**, 461–464.

10. Watanabe, S. 2010 Asymptotic equivalence of Bayes cross validation and widely applicable information criterion in singular learning theory. *J. Mach. Learn. Res.* **11**, 3571–3594.

11. Spiegelhalter, D. J., Best, N. G., Carlin, B. P. & van der Linde, A. 2002 Bayesian measures of model complexity and fit. *J. R. Stat. Soc. Ser. B Stat. Methodol.* **64**, 583–616. (doi:10.1111/1467-9868.00353)

12. Ripplinger, J. & Sullivan, J. 2008 Does choice in model selection affect maximum likelihood analysis? *Syst. Biol.* **57**, 76–85. (doi:10.1080/10635150801898920)
